# Smart Energy–Harvesting Coating for Moisture–Droplets Based on Ionic Diodes and Transistor–Like Structures

**DOI:** 10.1002/advs.202521476

**Published:** 2026-02-04

**Authors:** Liang Ma, Mengdi Liu, Yuxi Yang, Zehao Wang, Lan Shi, Limin Wu

**Affiliations:** ^1^ College of Smart Materials and Future Energy State Key Laboratory of Coatings for Advanced Equipment Advanced Coatings Research Center of Ministry of Education of China Fudan University Shanghai China

**Keywords:** droplet electricity generator, moisture electric generator, scalable coating, sustainable energy

## Abstract

The growing demand for distributed sustainable energy solutions has driven innovations in atmospheric moisture and droplet‐enabled electricity generation. This study introduces a dual‐mode moisture‐droplet energy‐harvesting coating (MDEC) that integrates a moisture electricity generator (MEG) and a triboelectric droplet electricity generator (DEG) into a single scalable coating system. By employing hybrid MXene‐bridged graphene oxide (GO) microspheres as the hybrid ink electrode and a fluorocarbon resin dielectric layer, the developed MDEC overcomes the limitations of traditional metal‐based electrodes that cannot be scaled for manufacturing and the inefficiency of single‐energy harvesting schemes in moisture environments. The MEG component achieves a voltage output of 0.85 V at 25% relative humidity through ion concentration gradient diffusion, whereas the DEG component has a peak power density of 36 W m^−2^ with a short‐circuit current of 301 µA and an open‐circuit voltage of 36.5 V. Modular integration of 360 units enables linear voltage scaling up to 301 V, successfully powering commercial LEDs and charging capacitor devices. This design offers a promising pathway for scalable low‐power electronics and Internet of Things (IoT) applications.

## Graphical abstract

A smart power‐generating coating is developed to harvest energy from both ambient humidity and impacting liquid droplets. The integrated system delivers sustained open‐circuit voltages of 0.85 V from moisture and up to 36 V from droplets. Its scalable coating architecture enables a continuous power supply for low‐power electronic devices.

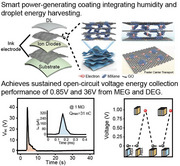

## 1 Introduction

The ever‐increasing demand for sustainable energy has inspired considerable research on ubiquitous environmental energy sources. Atmospheric moisture and sporadic water droplets that are widely distributed yet underutilized energy sources offer unique advantages for low‐frequency power generation [1, 2, 3, 4]. A moisture electricity generator (MEG) harnesses electrolyte concentration gradients induced by water molecule diffusion, whereas a droplet electricity generator (DEG) relies on liquid‐solid interfacial charge transfer during impact [5, 6, 7, 8]. Inspired by the principle of metal‐oxide‐semiconductor field‐effect transistors (MOSFETs), solid‐liquid contact ionization constructs circuits within the model to enable the separation of charges generated during droplet contact ionization to be instantaneously released through a “conductive channel” mechanism [8, 9]. This charge transfer process resembles the switching mechanism of a field‐effect transistor, significantly amplifying transient electrical signals compared to conventional electrostatic induction. Although integrating droplet‐based instantaneous harvesting with moisture‐based continuous harvesting would enable round‐the‐clock energy collection, representing a significant advance in the utilization of environmental water resources, few studies have attempted to combine the two mechanisms and approaches [10, 11, 12, 13]. The primary challenge lies in electrode construction, which cannot simultaneously accommodate both moisture and droplet energy harvesting. In addition, the use of noncoated electrodes severely limits scalable manufacturing.

On the other hand, polymeric coatings are widely used on the surfaces of almost all products. If energy‐harvesting coatings can be developed, then significant progress will be made in this field. While the development of energy‐harvesting coatings could revolutionize this field, current research remains insufficient in designing coating‐based electrode systems compatible with low‐temperature curing processes for moisture‐droplet energy harvesting applications. Specifically, Shen et al. [14] demonstrated a hybrid generator combining moist‐electric (MEG) and triboelectric (TEG) mechanisms for dual‐mode energy harvesting. However, the design relies on porous polytetrafluoroethylene (PTFE) membranes as dielectrics and Cu electrodes, with the multilayer electrode structure being difficult to prepare via coating methods and featuring a complex structural design. Similarly, the deformable wettable‐triboelectric nanogenerator hybrid structure proposed by Park et al. [15], while achieving an innovative breakthrough in compact design for self‐powered sensing, relies on mechanical deformation and is constrained by the inability to prepare scalable coating forms due to the hydrogel foam substrate. These collective shortcomings highlight the critical need for developing unified coating material systems enabling scalable droplet‐moisture energy harvesting technologies.

To address these issues, herein, we develop a dual‐mode coating system that integrates MEG and DEG functions through MXene‐enhanced hybrid ink electrode coatings. Optimized energy output for droplet and moisture energy harvesting through independent structural and functional design. Pinpoint‐controlled formation of surface functional groups (─O, ─OH, and ─F) plays a pivotal role in determining material properties, enabling 2D materials (e.g., MXene) to maintain high hydrophilicity while facilitating easy bonding with diverse substances [16, 17, 18]. This facilitates the combination of polymer coatings with conductive 2D material ink coatings for the scalable fabrication of energy harvesting systems. By employing a biomimetic polyelectrolyte film with graded ion transport channels, a 0.85 V output at 25% relative humidity (RH) and sustained droplet‐induced current generation were achieved. The MXene‐bridged architecture enables scalable fabrication through solution processing, overcoming the traditional limitations of metal‐dependent electrodes [6, 19]. Ion‐diode structure coatings featuring moisture‐resistant MXene‐PSSA polyanionic layers which maintained 92% voltage stability over 100 consecutive hours of cycling were combined with fully coated transistor‐like structures to achieve a power density of 36 W m^−2^ under intermittent droplet impact conditions.

## 2 Results and Discussion

### 2.1 Design and Application Scenarios of MDEC

**FIGURE 1:**
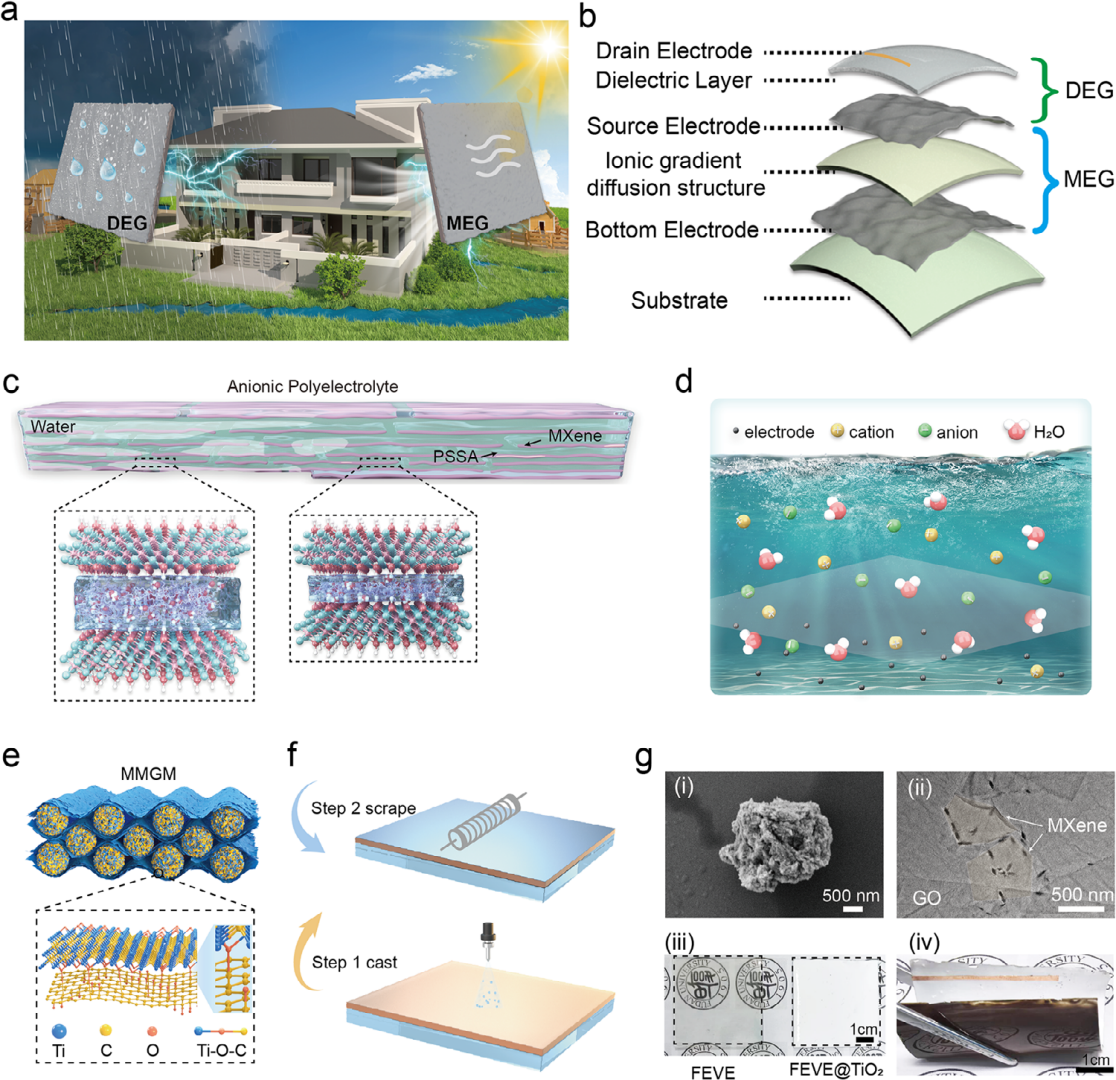
Structure and fundamental principles of MDEC. (a) Application scenario as a building coating. (b) Structural composition of MDEC. (c) Structure of an anionic polyelectrolyte. (d) Ion diffusion under moisture conditions. (e) Schematic diagram and model of the ink electrode with a sphere‐sheet structure. (f) Simplified construction process of DEG. (g) (i) Morphology of MGM microspheres, (ii) TEM image of MXene loaded on GO, (iii) Morphology comparison of the dielectric layer before and after TiO_2_ addition, (iv) Complete morphology image of MDEC.

A scalable, high‐performance dual‐mode MDEC was developed for building surfaces, enabling broader water and droplet energy harvesting. As shown in Figure 1a, when the coating adheres to building surfaces such as conventional architectural coatings, it collects droplet energy during rainfall and generates electricity using moisture within the walls or atmospheric humidity on sunny days. Owing to their specialized application context, conventional metallic and noncoated electrodes cannot be prepared in this coating form. However, this approach is feasible when 2D material ink electrodes are used. The composition of this coating system, where the substrate can be composed of various materials including paper, polyethylene terephthalate (PET), and polydimethylsiloxane (PDMS), is shown in Figure 1b. Previously proposed MXene‐bridged MXene‐GO microspheres (MMGM) were used to construct hybrid 2D material inks as electrode layers for MEG and DEG [20]. As schematically illustrated in Figure S1, delaminated Ti_3_C_2_T_x_ was prepared via the minimally intensive layer delamination (MILD) method [21]. Through a homogeneous reaction with a GO aqueous solution, the Ti_3_C_2_T_x_ sheets effectively bridged and crosslinked with GO via nucleophilic substitution and dehydration reactions, resulting in the formation of Ti─O─C bonds under hydrothermal conditions and hydrogen bonding interactions at room temperature [22]. The layered structure of MXene‐GO effectively suppresses the self‐stacking phenomenon of MXene sheets, significantly increasing the overall charge density [23]. Energy dispersive spectrometer (EDS) mapping (Figure S2) revealed a homogeneous elemental distribution, confirming the uniform cosynthesis of the composite materials. X‐ray photoelectron spectroscopy (XPS) analysis (Figure S3) revealed characteristic bonding states in MMGM: The Ti 2p spectra exhibited four peaks at 453.8, 454.2, 455.3, and 457.3 eV, whereas the C 1s spectra showed four components at 281.4, 284.5, 284.8, and 285.2 eV. The detection of Ti─O─C covalent bonds through these spectral features confirmed successful interfacial bridging between the titanium and graphene oxide carbon of the MXene. Scanning electron microscopy (SEM) characterization (Figure S4) revealed a hierarchical sphere‐sheet architecture in the MMGM composite, where the MXene‐GO nanospheres generated through freeze‐drying formed an interconnected porous network. This 3D configuration was shown to increase electrical conductivity while providing accessible active sites for charge storage. The cross‐linking process between aerogel microspheres and MXene flakes was evidenced by the transformation from 2D planar sheets to 3D interconnected frameworks. Electrochemical measurements indicated that the structural integrity of the ink electrode was maintained when it was deposited on PDMS substrates, demonstrating its compatibility with flexible MEG device fabrication requirements.

The typical channel structure and operating principle of the anionic polyelectrolyte, which consists of MXene dispersed as a filler within PSSA (a mixture of poly(sodium p‐styrene sulfonate) (PSS) and polyvinyl alcohol(PVA)), are shown in Figure 1c. The ion gradient diffusion structure incorporates a cationic polyelectrolyte layer formed by PDDA and an anionic polyelectrolyte layer composed of MXene and PSSA, creating a natural ion gradient diffusion pathway. An MMGM layer was deposited onto the ion diffusion and served as both the top electrode for the MEG and the source electrode (SE) for the DEG, enabling the preparation of the ink electrode via drop coating or spraying. The dielectric layer (DL) employs novel fluorocarbon resin FEVE (fluoroolefin‐alkyl vinyl ether/ester copolymer), which can be cured at room‐temperature curability. As a common dielectric‐enhancing material, rutile‐type titanium dioxide (TiO_2_) demonstrates advantages in dielectric properties and cost‐effectiveness (Table S1). By incorporating TiO_2_ nanoparticles as a dielectric‐enhancing filler into the system, FEVE is colored while simultaneously improving its dielectric properties, enabling broad application in coating formulations. When water molecules diffuse into the interlayer space of the polyelectrolyte, the transferred electrons and the chemically absorbed ions on the solid surface form an electrostatic field, resulting in the formation of an electrostatically induced opposite charge of equal magnitude on the lower electrode. (Figure 1d). With the process of preparing aerogel microspheres shown in Figure S5, the aerogel microspheres obtained through liquid nitrogen rapid freezing followed by low‐temperature freeze‐drying exhibit a layered arrangement structure. They form the microstructure of the ink electrode by bridging MXene via Ti─O─C covalent bonds (Figure 1e). The entire coating structure can be constructed through simple spray or scrape coating methods (Figure 1f). Figure 1g illustrates the entire process from microsphere preparation to film formation: (i) shows the morphology of MGM; (ii) displays the TEM morphology of GO‐loaded MXene; (iii) compares the light transmittance of FEVE coatings before and after adding TiO_2_ as a coloring filler; (iv) presents the morphology of the final composite coating. DEG can be prepared (including the entire system) through simple drop coating or spraying, as shown in Figure 1f. When the droplets contact the dielectric layer, a point bilayer forms to facilitate charge transfer and exchange, thereby disrupting the charge equilibrium [24]. The prepared coating exhibits excellent mechanical properties and stability, including the key characteristic of room‐temperature film formation, thereby enabling its large‐scale application. Figure S6 illustrates the charge distribution within the coating during the liquid droplet's landing and sliding process. Notably, the appearance of the FEVE resin changed from transparent before the addition of TiO_2_ to white after its addition, indicating that this resin material can be modified with pigments to adapt to diverse application requirements (Figure S7).

### 2.2 Working Principle of the DEG Module

**FIGURE 2:**
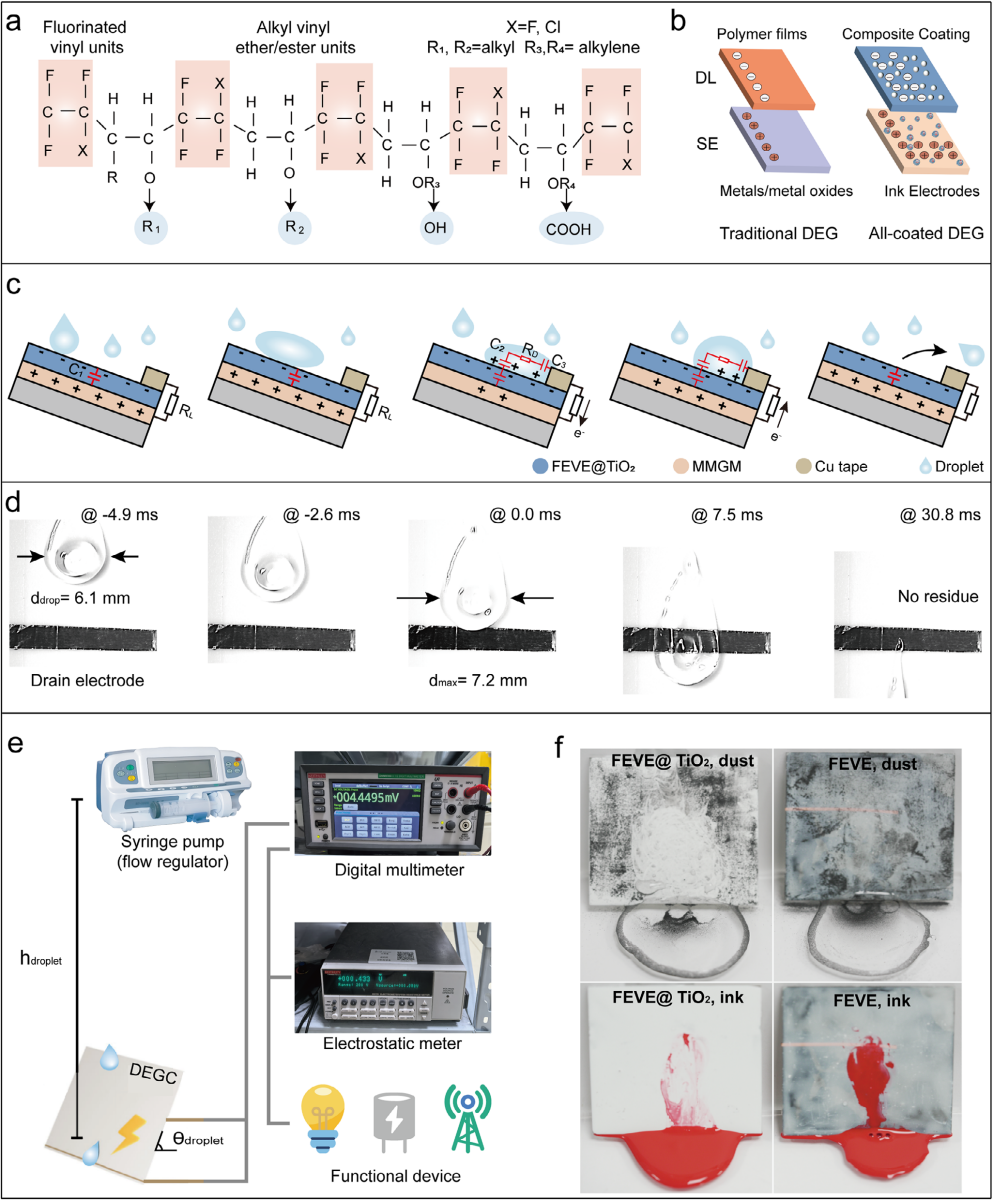
Preparation of DL and the mechanism of DEG. (a) Structure of FEVE. (b) Comparison of the ink electrode and the metal electrode. (c) Mechanism of DEG and time trajectory of sliding process (d). (e) Schematic of the droplet energy harvesting process. f. Comparison of self‐cleaning and anti‐soiling results between FEVE@TiO_2_ and FEVE.

FEVE is a room‐temperature curable fluorocarbon resin that exhibits superior weatherability properties (water contact angle >100°), a low friction coefficient, and self‐cleaning properties [25]. Its molecular structure (Figure 2a) comprises fluorinated vinyl units and alkyl vinyl ether/ester units, achieving room‐temperature crosslinking with isocyanates via the introduction of alkyl/hydroxyl/carboxyl groups.

By doping TiO_2_ into the FEVE emulsion as a dielectric enhancer (Figure 2b), we simultaneously enhanced the dielectric performance and increased hydrophobicity, addressing the functionalization challenges of traditional DL (Figure S8). Unlike conventional membrane‐metal electrode assemblies, this work establishes a fully coated dual‐electrode system: the SE utilizes an MMGM layer on the PDDA, while the drain electrode (DE) employs linear Cu conductive tape. This design converts interfacial effects into bulk effects through electrostatic induction–triboelectric charge synergy (Figure 2c), circumventing interfacial shielding while reducing structural complexity [8]. A closed‐loop circuit is formed via copper tape connections to a load resistor R_L_.

Upon impacting the triboelectric electrode surface, the droplet's power generation process is divided into five stages on the basis of its motion state: (i) When the droplet contacts the DL surface enriched with electrons, cations within the droplet combine with DE, forming a pseudocapacitor C_1_ at the contact interface. (ii) As the droplet flows along the DL surface, the total charge in the system remains unchanged. Charge transfer requires reaching the top electrode to enable external circuit charging and maintaining the system in a closed state. (iii) When the droplet reaches the DE, new pseudocapacitors form between the DL and droplet C_2_, and between the DE and droplet C_3_. This creates a closed‐loop circuit with the droplet resistance R_D_, switching the system to a conductive state. Subsequently, electrons previously stored in C_1_ rapidly flow toward the SE. (iv) During droplet contraction, electrons return to the DE, and DL recharges via C_2_ and C_3_. The droplet's expansion and contraction processes demonstrate the charging and discharging characteristics of pseudo capacitors. (v) When droplets continuously flow out from the DE, the power generation cycle terminates. The charge exchange process at the molecular level, as shown in Figure S9, explains the charge exchange mechanism between FEVE and water molecules and the generation of electrostatic energy. Figure 2d documents the entire process corresponding to droplet sliding, revealing that at the point of maximum diameter, the droplet contacts the DE and exhibits prolonged sliding time due to the hysteresis effect. Closed‐loop circuit with the droplet resistance R_D_, switching the system to a conductive state. Subsequently, electrons previously stored in C_1_ rapidly flow toward the SE. (iv) During droplet contraction, electrons return to the DE, and DL recharges via C_2_ and C_3_. The droplet's expansion and contraction processes demonstrate the charging and discharging characteristics of pseudo capacitors. (v) When droplets continuously flow out from the DE, the power generation cycle terminates. Figure 2d documents the entire process corresponding to droplet sliding, revealing that at the point of maximum diameter, the droplet contacts the DE and exhibits prolonged sliding time due to the hysteresis effect.

The electrical measurements were conducted by using an electrometer (integrated with MATLAB) for high‐precision current/charge measurements, and a digital multimeter for voltage/current recording (Figure 2e). The equivalent circuits for the MEG and DEG energy harvesting processes are shown in Figure S10. Furthermore, DL has a self‐cleaning and antifouling ability from outdoor contamination owing to its strongly hydrophobic surface, which effectively prevents the reduction in triboelectric performance (Figure 2f). To assess this issue, fine sand grains were employed as dust, and inked water was used to easily identify the residue on the surface. When water is dropped on the FEVE surface, it tends to remain on the surface, hindering effective cleaning in the presence of dust and leaving residue on the surface. In contrast, when water is dropped on the surface, the droplets easily roll off, carrying away the powder; in this case, the DL can be effortlessly cleaned with the droplets.

### 2.3 Performance Optimization and Output Performance of the DEG

**FIGURE 3:**
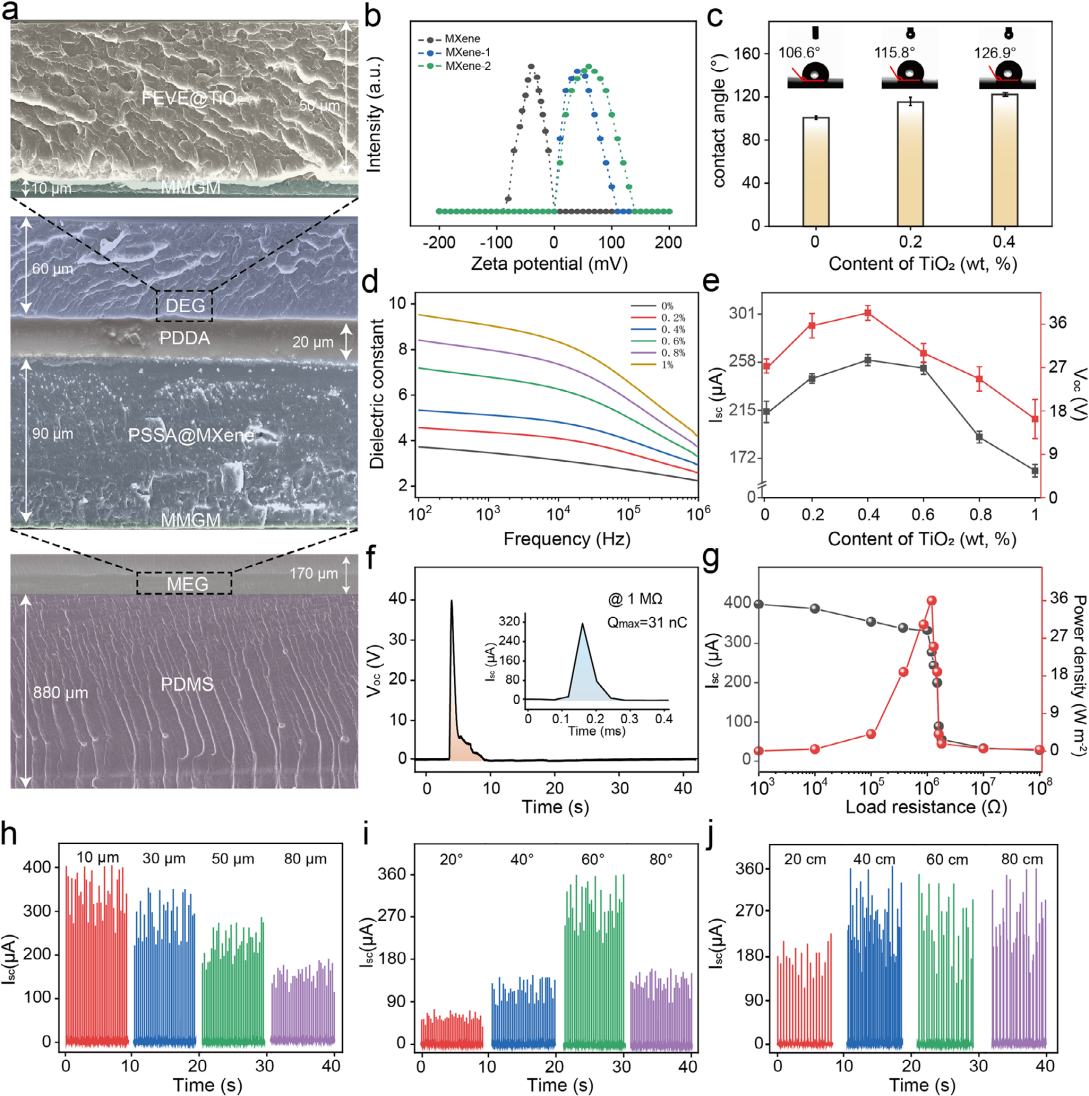
Droplet energy harvesting performance. (a) MDEC overall structural cross‐section diagram. (b) Zeta potential distribution of MXene before and after PDDA anodization. (c) Effect of TiO_2_ content on the contact angle of the dielectric layer. (d) Effect of different TiO_2_ loading levels on the dielectric constant of the dielectric layer. (e) Effect of TiO_2_ content on droplet energy harvesting performance. (f) V_oc_ and I_sc_ waveforms at optimal output. (g) I_sc_ and peak power density corresponding to different loads (h) The influence of FEVE thickness, coating tilt angle (i), and droplet height (j) on output performance.

The cross‐sectional morphology of the dual‐mode coating, as shown in Figure 3a, clearly illustrates the structural composition of both the MEG and DEG components. PDMS, characterized by thermal stability, cold resistance, low surface tension, and viscosity‒temperature stability, forms the substrate of the entire system. Overlying this is the MEG section, which is primarily composed of a triboelectric electrode layer, a polyanionic layer composed of PSSA and MXene, and a PDDA polycationic layer. The DEG section is deposited onto the MEG layer, primarily comprising the MMGM electrode layer and a dielectric layer.

As the entire system can be rapidly fabricated using common coating preparation methods such as dip coating, spin coating, and spray coating, without the need for complex processes. The prepared functional layers exhibit high consistency in morphology and thickness uniformity (Figure S11). As shown, cross‐sections of the electrode, ion diffusion, and dielectric layer—key components of the system—were selected, and thickness measurements were performed at randomly sampled points across ten specimens. The results demonstrate that all functional layers possess high consistency. MXene nanosheets exhibit hydrophilic surfaces and are terminated with abundant functional groups (═O, ─OH, and ─F). These groups act as charged units, imparting a negative charge to the pristine MXene nanosheet surface [17]. Negatively charged MXene nanosheets were first dispersed into a solution of the cationic polymer PDDA via probe ultrasonication to obtain nanosheets with positive surface charge polarity. This further enhances the ability of MXene to attract negative charges when used as an electrode, thereby improving its conductive properties [26].

As shown in the zeta potential distribution in Figure 3b, the zeta potential of the MXene modified with 2 wt.% PDDA shifted from an initial value of −42 to +46 mV. The hydrophobicity of the dielectric layer also significantly changed after the addition of TiO_2_, with the contact angle increasing from 106.6° without TiO_2_ to 126.9° (Figure 3c). Since the charge transfer at the liquid‐solid interface highly depends on the sliding speed of the droplet on the hydrophobic surface, enhancing the hydrophobicity facilitates the droplet's sliding speed of the droplet on the dielectric layer, thereby accelerating charge migration [27]. Testing the changes in the dielectric constant induced by varying the TiO_2_ mass fraction reveals that the dielectric constant gradually increases with increasing TiO_2_ content (Figure 3d).

However, higher dielectric constants do not necessarily correlate with enhanced DEG output performance. This is primarily because excessive TiO_2_ loading leads to agglomeration and reduced hydrophobicity (Figure S12). As shown in Figure 3e, when the TiO_2_ loading was 0.4 wt.%, the DEG exhibited a short‐circuit current (I_sc_) of 301 µA and an open‐circuit voltage (V_oc_) of 36.5 V. Under an external load of 1.0 MΩ, the maximum charge transfer per droplet reached 31 nC (Figure 3f), with the DEG exhibiting a peak power density of approximately 36 W m^−2^ (Figure 3g). Furthermore, the output performance of the DEGs correlated with the dielectric layer thickness (Figure 3h), contact angle (Figure 3i), and droplet impact height (Figure 3j). The effects of DL thickness, film tilt angle, and droplet drop height on V_oc_ and transferred charge quantity(Q) are presented in Figure S13. It can be observed that increasing DL thickness suppresses output performance, while both tilt angle and droplet drop height influence sliding speed and contact time. Consequently, the overall trend shows that output performance increases with greater contact angle and height. As previously stated, the output performance of the DEG is closely related to the pseudo capacitance at the interfaces. The interface capacitances (C_1_ and C_2_) between the DL and the SE layer, as well as between the DL and the DE, are determined by the dielectric constant (ε) and thickness (d). Therefore, the dielectric constant and the thickness of the DL are crucial for optimizing the performance of the DEG. Their relationships are described by Equations (1) and (2):

(1) $$C_{1}=\frac{\varepsilon_{\mathrm{FEVE}}S_{\mathrm{FEVE}}}{d_{\mathrm{FEVE}}}$$

(2) $$C_{2}=\frac{\varepsilon_{\mathrm{water}}S_{\mathrm{FEVE}}}{d_{\mathrm{EDL}}}$$

where ε_FEVE_ denotes the dielectric constant of the FEVE coating, S_FEVE_ represents the surface area of the FEVE coating, d_FEVE_ and d_EDL_ denote the thickness of the FEVE coating and the thickness of the electric double layer (EDL), respectively. When ε_FEVE_, S_FEVE_, and d_EDL_ are fixed, since the thickness of pseudocapacitance C_1_ is typically several orders of magnitude greater than C_2_, appropriately reducing d_FEVE_ is more advantageous for increasing the controlling factor C_1_. The accumulation of the triboelectric charge in the DEG can be expressed by Equation (3):

(3) $$Q\;=\;CV_{\mathrm{Tri}}$$

where Q represents the accumulation of triboelectric charge, C denotes the capacitance between the source electrode and the dielectric layer, and V_Tri_ represents the voltage amplitude of the triboelectric wave. Therefore, the triboelectric charge accumulation can finally be calculated as follows:

(4) $$Q=\frac{\varepsilon_{\mathrm{FEVE}}S_{\mathrm{FEVE}}}{d_{\mathrm{FEVE}}}V_{\mathrm{Tri}}$$

when the V_Tri_ of the liquid droplet occurs, many charges on the dielectric layer recombine with opposite charges in the SE. Therefore, the thickness of the FEVE coating critically affects the DEG output performance. However, constrained by the preparation process using the coating method, 30 µm represents the minimum achievable layer thickness. Considering subsequent application scenarios, we determined the overall preparation thickness of the FEVE coating to be 50 µm.

The variation in the dielectric constant (ε) variation among different droplets also affects the quantity of charge transferred. We therefore compared the output performance of various droplets. As shown in the comparative diagram (Figure S14), deionized water, dilute saline solution, and rainwater all generated outputs, with notably superior performance observed in rainwater during testing. Additionally, the pH of the droplet also affects output performance (Figure S15). If the droplet's pH exceeds 3, it becomes positively charged, while the dielectric material surface carries a negative charge, leading to an increase in contact charge. These results demonstrate the practical feasibility of using the DEG in real‐world applications.

### 2.4 Structure and Mechanism of the MEG Module

**FIGURE 4:**
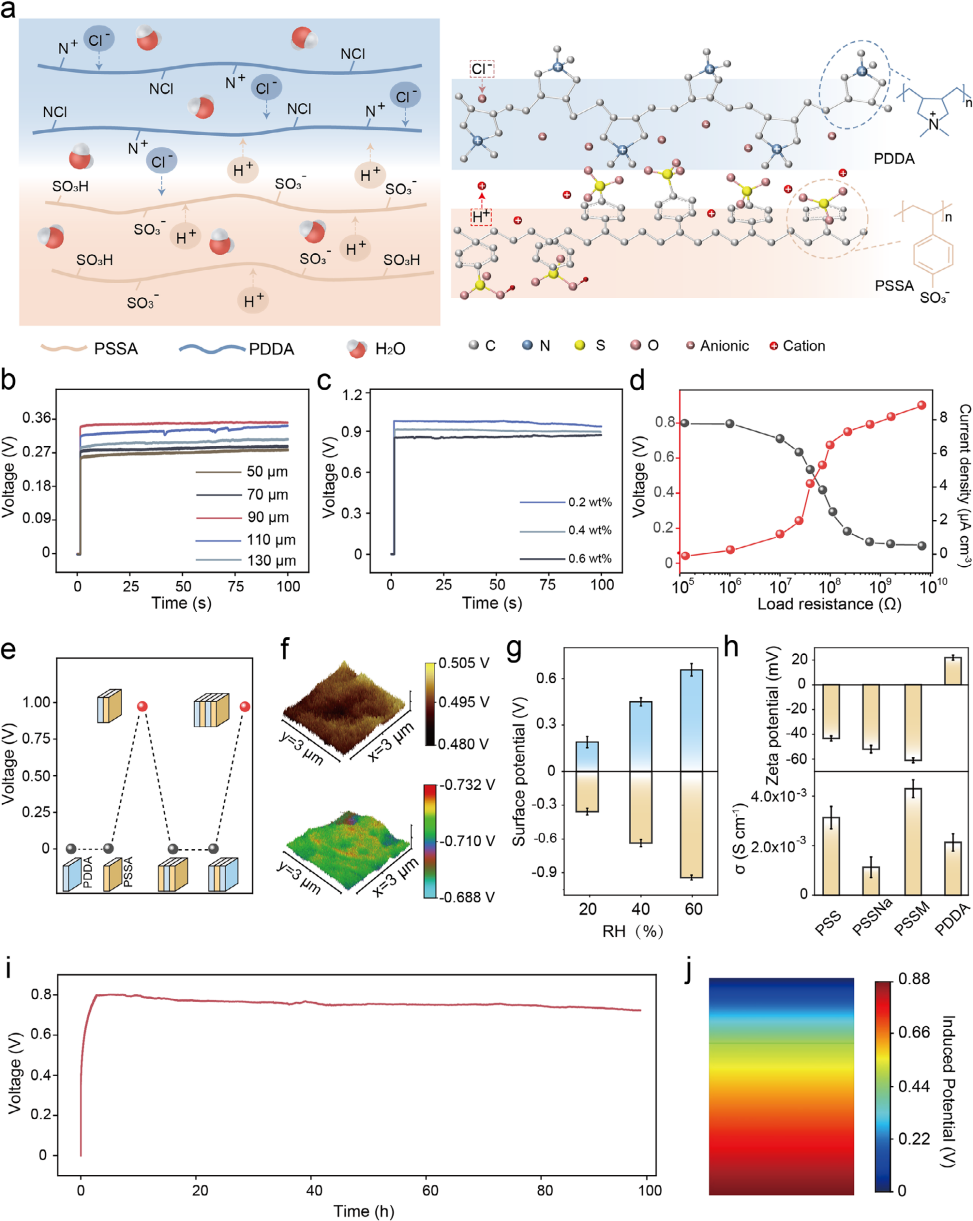
The performance and mechanism of MEG. (a) Schematic diagram of the moisture‐driven power generation principle in a double‐layer coating. (b) The influence of PSSA layer thickness on performance. (c) The influence of MXene addition on output performance. (d) Voltage and volumetric current density output of the MEG with variable electric resistance. (e) Induced voltage with different stacking layers of PDDA and PSSA components. (f) Relative surface potentials of the PDDA and PSSA layer under 25% RH and 25°C. (g) Relative surface potential of BPF bilayers under varying RH. (h) Zeta potential (ζ) of polyelectrolyte aqueous solutions (0.1 mg mL^−1^, pH ≈ 7.0) and ionic conductivity (σ) of polyelectrolyte films were measured by electrochemical impedance spectroscopy at 75% RH (25°C). (i) The voltage output of the MEG is sustained for 100 h under atmospheric conditions of ∼25% RH and 25 ± 5°. (j) Calculated induced potential distribution profile along the thickness direction of MEG coating in steady state.

An ion concentration gradient diffusion coating was constructed by combining PDDA with PSSA doped with MXene (Figure 4a). The rationally incorporated PVA component endows the PSSA film formed from PSS and PVA with outstanding flexibility [28]. As the coating spontaneously adsorbs water molecules from humid air over extended periods, negatively charged Cl^−^ ions dissociate within the PDDA layer while positively charged H^+^ ions dissociate within the PSSA layer. A concentration gradient of ions naturally forms, driving outward diffusion and inducing a potential difference (Figure 4a). After being modified with PDDA, the MXene surface is covered by quaternary ammonium cations (─N^+^(CH_3_)_3_) through electrostatic adsorption, with some original ─OH groups protonated (─OH_2_^+^). The presence of more cations in the surface functional groups results in the release of positive charges, thereby enhancing the output performance.

The test results indicate that when the MMGM serves as the electrode, altering the thickness of the PSSA layer affects the moisture collection performance, with the output performance peaking at a thickness of 90 µm (Figure 4b). As shown in Figure 4c, the addition of modified MXene further enhances the moisture collection performance, yielding a maximum V_oc_ of approximately 0.9 V when 0.2 wt.% MXene is incorporated. At an external load of 60 MΩ, the MEG achieved a peak volumetric power density of 1.1 µW cm^−3^ (Figure 4d).

Comparative analysis (Table S2) reveals that moisture power generation efficiency is generally lower under low RH conditions, presenting a significant challenge that current research must address and overcome. Monolayer polyelectrolyte films (PDDA/PSSA) failed to generate electricity because of a lack of heterostructures (Figure 4e), while four‐layer stacks (PDDA/PSSA/PDDA/PSSA) exhibited bilayer‐like performance, highlighting the role of ion diffusion. Upon prolonged humidity exposure, spontaneous water adsorption sustains ion dissociation and diffusion, thereby maintaining the voltage output (Figure 4f,g). KPFM measurements revealed an ∼1.2 V surface potential difference between the PDDA (∼0.5 V) and PSSA (∼−0.7 V) layers posthydration (Figure 4f). Increased RH amplified this potential difference (Figure 4g), confirming increased H^+^/Cl^−^ dissociation and heterogeneous charge distribution. Power generation relies on three factors: spontaneous water absorption, ion dissociation/distribution, and effective ion diffusion within the MEG.

Zeta potential (ζ) and ionic conductivity (σ) analyses (Figure 4h) confirmed the correlations of the voltage/current with the surface charge capacity and ion mobility. A continuous 100 h test under 25% RH resulted in a stable ∼0.8 V output (Figure 4i). Performance of MEG energy harvesting under different RH conditions (Figure S16) indicates that higher RH correlates with enhanced moisture output performance, which is closely related to the diffusion of water molecules. At the same time, air temperature significantly impacts the performance of moisture‐based power generation (Figure S17). Impressively, MEG operates effectively across a broad temperature range from −18°C to 60°C (25% RH), and elevated temperatures actually enhance its output performance. An MEG voltage of 0.85 V was predicted on the basis of numerical simulations coupling the Nernst–Planck and Poisson equations; this prediction aligns with the experimental data, validating the mechanism. The MEG maintains stable electrical output in prolonged humidity environments, ensuring reliability for practical applications.

### 2.5 Scalable Integration of MDEC

**FIGURE 5:**
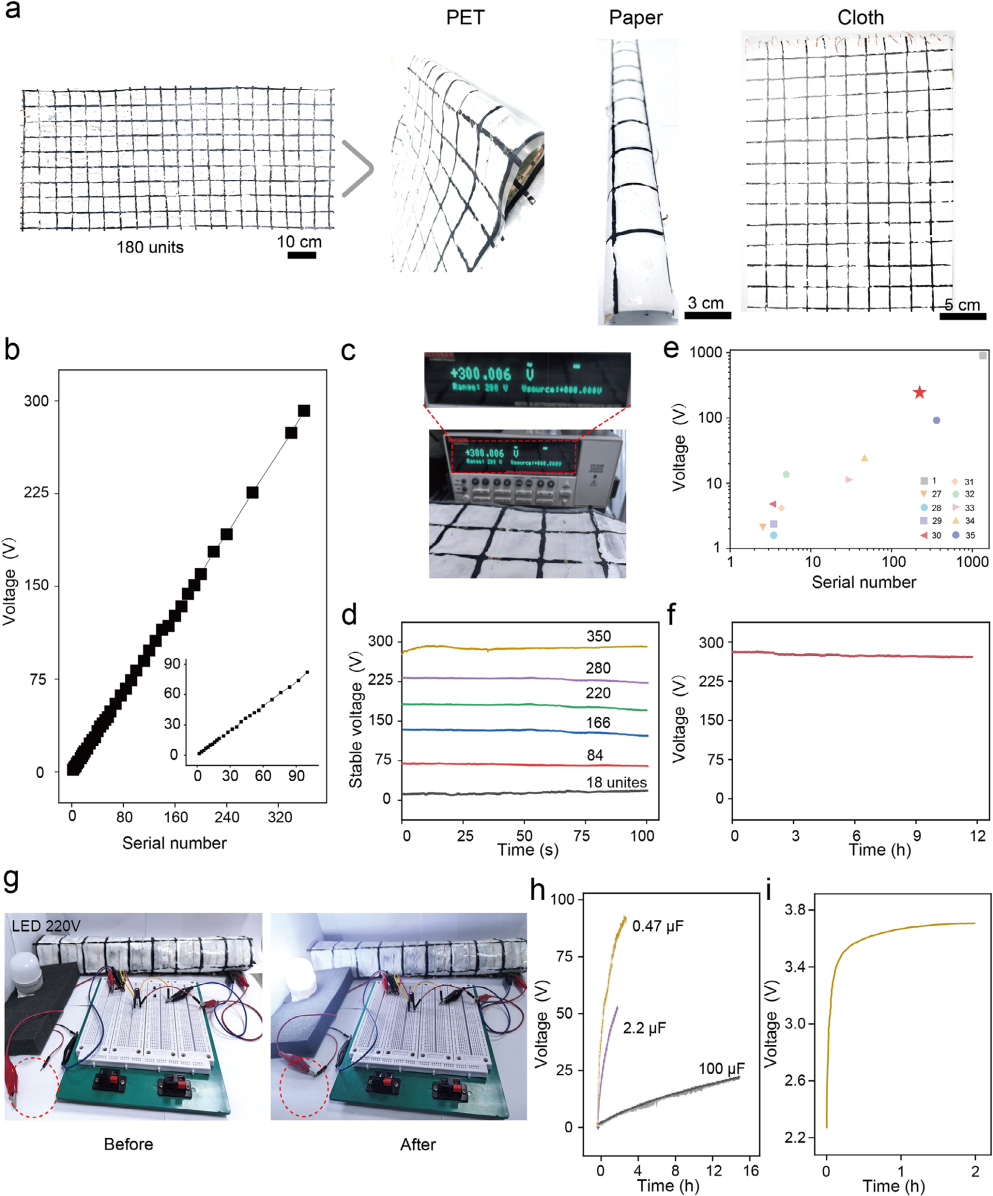
Integration of MDEC units. (a) Photographs of flexible integrated devices on different substrates, including PET, paper, and cloth. (b) Voltage output curves for MDEC units with different serial numbers. The inset shows an enlarged view of serial numbers 1–90. (c) A photograph of an integrated device array capable of generating 300 V under ambient conditions (25% RH, 25°C). (d) Stable voltage signals from integrated devices can be connected in series with 18, 84, 166, 220, 280, and 350 units. (e) Systematic performance comparison of reported integrated devices based on various materials. (f) The voltage output of an integrated device with 360 units in series was tested for 12 h (30% RH, 25 ± 5°C). (g) A lamp bulb (10 W) driven by integrated MDECs. (h) Voltage–time curves of commercial capacitors of varying capacitance. (i) Voltage–time curve of a lithium‐ion battery with a capacity of 0.3 mA h charged by integrated MDECs.

Scalable integrated power generation technology is key to achieving high‐performance electricity output under environmental conditions. Here, a large‐scale integration strategy was developed, enabling the preparation of individual power‐generating units on a single substrate through a modular, series‐connected coating process. The integrated power‐generating coating is shown in Figure 5a. In the experiments, 180 power‐generating units were integrated onto a PET plastic substrate, and this fabrication method is reproducible. Substrate selection also allows for the use of diverse components, including polyethylene terephthalate, paper, and fabric. The voltage output of the integrated device increases linearly with the number of series units, as shown in Figure 5b. The inset illustrates that linear output scaling is achievable even with a small number of series units, confirming the scalability of the MDEC. A coating integrated from 360 MDEC modules can deliver approximately 300 V (Figure 5c). The stable voltage outputs achievable with varying numbers of MDEG modules in series are shown in Figure 5d, whereas the output voltages generated by different series configurations are compared in Figure 5e [29, 30, 31, 32, 33, 34, 35, 36, 37, 38].

The amplification and effective utilization of moisture energy through integrated approaches represent the current mainstream research trend (Table S3). A stable output was also demonstrated during a continuous 12 h test (Figure 5f), in which a 10 W commercial LED bulb operating at successfully powering a 10 W commercial LED bulb operating at 220 V was successfully powered (Figure 5g). As shown in Figure 5h, the electrical energy supplied by the MDEC integrated device can be stored in commercial energy storage equipment without the need for an additional rectifier. Capacitors of 0.47, 2.2, and 100 µF can be charged to 90, 55, and 20 V, respectively. With this integrated MDEC device, lithium‐ion batteries can also be charged, as shown in Figure 5i, achieving a charging voltage of approximately 3.5 V in approximately 2 h. The behavior of the MDEC integrated coating device under temperature and humidity influences exhibits similar patterns to those observed in single structural units (Figure S18). Results indicate significantly improved charging efficiency under high humidity conditions (25°C, 85% RH), enabling a 100 µF commercial capacitor to reach 32 V within 6 h. In contrast, under low humidity conditions (25°C, 25% RH), the voltage reached only 12 V. The coating‐based dual‐mode energy harvesting system not only overcomes the limitations of single‐mode devices but also provides a scalable pathway toward practical applications such as powering building‐integrated electronics, IoT devices, and distributed sensors.

## 3 Conclusion

In summary, we demonstrated a breakthrough in dual‐mode energy harvesting by combining moisture and droplet‐driven mechanisms within a unified coating system. Key innovations include the use of MXene‐functionalized GO microspheres to mitigate electrode restacking, the optimization of FEVE@TiO_2_ dielectric layers to achieve enhanced triboelectric output, and the application of a modular integration strategy to enable scalable voltage amplification. The experimental results validate the ability of the MEG to generate 0.85 V under low humidity (25% RH) and the high‐power density of the DEG (36 W m^−2^), with the integrated systems achieving 301 V output. The self‐cleaning properties, mechanical flexibility, and compatibility of the coating with diverse substrates (e.g., PET and fabric) underscore its practicality for real‐world deployment. Furthermore, the system's ability to charge capacitors and lithium‐ion batteries highlights its potential for autonomous IoT devices. Future research should focus on long‐term environmental durability and further optimization of ion diffusion kinetics to broaden its applicability in sustainable energy systems.

## 4 Materials and Methods

### 4.1 Materials

Ti_3_AlC_2_ (MAX) powders were purchased from Jilin 11 Technology Co., Ltd. GO aqueous dispersions were purchased from Hangzhou Gaoyan Technology Co. The FEVE emulsion was purchased from Changzhou Hongfu New Material Co., PDMS was purchased from Sigma–Aldrich Chemical Co., Ltd. Lithium fluoride (LiF), PSS, PVA, and TiO_2_ were purchased from Aladdin Industrial Corporation. Hydrochloric acid (HCl) was obtained from Sinopharm Chemical Reagent Co., Ltd. (China). All chemical reagents were of analytical grade and did not require further purification.

### 4.2 Preparation of Monolayer Fluorinated MXene Nanosheet Solutions

Ti_3_AlC_2_ precursor was etched in 40 mL of 9 m HCl containing 4 g LiF at 35°C for 24 h under continuous stirring. The resulting dark green MXene (Ti_3_C_2_F) suspension underwent sequential centrifugation (3500 rpm, 5 min) and five cycles of washing with deionized water until a neutral pH (≈7) was achieved. The purified MXene was redispersed in water and sonicated in an ice‐bath sonicated for 1 h to exfoliate the nanosheets. Subsequent centrifugation (3500 rpm, 1 h) yielded a colloidal supernatant with a MXene concentration of 1.0 mg/mL. The monolayer nanosheets were collected by vacuum drying at 60°C for 12 h, following established exfoliation protocols. The morphology of the MAX phase before exfoliation and the exfoliated MXene are shown in Figure S19.

### 4.3 Fabrication of MGM

Porous GO and MXene microspheres were synthesized by low‐temperature spray freeze‐drying. First, GO and MXene were separately mixed into 50 mL of aqueous dispersion at a concentration of 10 mg mL^−1^ and stirred at 500 rpm for 30 min. Then, the obtained mixed dispersion was atomized by a commercial motorized sprayer atomizing nozzle, sprayed directly into liquid nitrogen, and then immediately frozen. Finally, the frozen droplets were placed in a vacuum freeze dryer and dried at 0.1 Pa for 72 h to obtain porous composite microspheres. The surface of the freeze‐dried aerogel microspheres is wrinkled, as shown in Figure S20, endowing them with an ultrahigh specific surface area, abundant surface functional groups, and electron migration pathways. This configuration provides favorable conditions for charge migration and ion diffusion. Raman spectroscopy (Figure S21a) revealed the A_1g_ (197/719 cm^−1^) and E_g_ (367 cm^−1^) vibrational modes of the MXene. GO showed D/G bands at 1330/1598 cm^−1^, whereas MGM exhibited a 17 cm^−1^ G‐band blueshift (1615 cm^−1^) and an increased I_D_/I_G_ ratio (0.9→1.2→1.5 in MMGM), confirming Ti─O─C bonding. MMGM displayed a further G‐band shift (1620, 22 cm^−1^) with new peaks at 838/1064 cm^−1^ and enhanced C─O/C─H/C═O signals (Figure S21b). XRD analysis revealed that the (002) peak at 6.5°, shifted to 10.8° in the composites, indicating reduced interlayer spacing (Figure S21c). The data in Figure S21 reveals MXenes with hexagonal features adhering to the wrinkled surface of the GO nanosheets. High‐resolution TEM characterization (Figure S22a) confirms the covalent crosslinking of MXene nanosheets with MGM to form a heterostructure. The interplanar spacing of the free MXene nanosheets is approximately 0.257 nm, whereas the spacing increases to 0.376 nm for the MXene nanosheets within the freeze‐dried microspheres crosslinked with GO. The disordered amorphous state of GO in the microsphere SAED pattern (Figure S22b) resulted in blurred diffraction rings. In contrast, the (100) crystal plane of the MXene nanosheets exhibited a characteristic diffraction ring with a 1/r value of 3.72 nm^−1^.

### 4.4 Fabrication of MMGM

MGM‐enhanced coated electrode layers were synthesized by hydrothermal synthesis. Specifically, 60 mg of the synthesized MGM was first added to 15 mL of 5 mg mL^−1^ MXene solution with vigorous stirring at 600 rpm for 0.5 h, and then transferred to a PTFE‐lined high‐pressure reactor with a volume of 30 mL. Next, the reactor was placed in an oven at 180°C for 6 h to obtain the MMGM solution with a self‐assembled sphere‐sheet structure. The obtained MMGM solution was repeatedly centrifuged at 3500 rpm, and the product was subsequently washed with ethanol and deionized water to a pH of approximately 7 and then centrifuged to obtain the precipitate. Finally, MMGM powder was obtained after drying in a vacuum oven at 60°C for 12 h. Test data or results related to the structural integrity of ink electrodes deposited on PDMS substrates were verified through cyclic voltammetry and constant current charge‐discharge (GCD) stability tests (Figure S23a). The data are based on the coating system after 1000 repeated flexing cycles. Cyclic voltammetry (CV) scans for an assembled coating system based on MMGM‐PDMS are shown in Figure S23b. For potential scan rates between 10 and 350 mV s^−1^, the coulombic efficiency was close to 100%. From the GCD curves, the MMGM‐PDMS sheets provided a volumetric capacitance of 465 F cm^−3^ at the current density of 1.0 A cm^−3^, and retained 75.3% of this capacitance for a current density of 4.3 A cm^−3^ with the coulombic efficiency of ∼95%. The results indicate that the MMGM‐PDMS composite exhibits excellent structural integrity.

### 4.5 Fabrication of MDEC

Coated electrode layers were synthesized by drop‐casting. Specifically, a layer of PDMS was spin‐coated on a 5 × 5 cm^2^ PET using a Sylgard 184 silicone elastomer substrate, the PDMS precursor was spin‐coated at a speed of 4000 rpm for 60 s, and then dried at 60°C for 0.5 h to allow for complete curing. Oxygen plasma treatment was applied to the PDMS substrate with O_2_ (5 sccm gas flow), using a power plasma treatment of 50 W for 5 min. This plasma treatment resulted in a completely hydrophilic PDMS surface. Afterward, aqueous dispersions of MMGM at a concentration of 10 mg mL^−1^ were drop‐cast onto the PDMS substrate to construct the bottom electrode. PVA was dissolved in deionized water at 95°C with continuous stirring for 6 h. MXene/PVA mixtures were prepared by mixing the polyvinyl alcohol solution into MXene dispersions under magnetic stirring, followed by ultrasonic treatment. An aqueous PSS dispersion (5 wt.%) was uniformly mixed with an MXene/aqueous polyvinyl alcohol (PVA) solution (10 wt.%) and drop‐coated onto the MMGM. By controlling the mass ratio of PVA to PSS to 17%, a PSSA film was obtained. A 35 wt.% PDDA solution was subsequently sprayed onto the PSSA film surface to form a PDDA coating. This established an ion concentration gradient diffusion structure. A layer of MMGM was sprayed onto the PDDA surface to serve as the source electrode. A mixed emulsion of FEVE and TiO_2_ was prepared on the MXene using a squeegee coating strategy. Finally, a 2 mm wide Cu tape was adhered to the surface of the FEVE to obtain the MDEC.

### 4.6 Measurements and Characterizations

The ionic concentration of the tap water used in this experiment was 4.6 mm, and the rainwater was obtained by natural collection and had an ion concentration of approximately 5.8 mm. The electrical properties of the films were tested with a Tektronix nanogenerator software 6517b electrostatic meter with MATLAB and a digital multimeter DMM6500. In all the measurements, the environmental temperature and RH were maintained at approximately 25°C and 60%, respectively. Raman spectra were measured using an apparatus from Renishaw Laser using a laser excitation of 632 nm. FTIR spectra were obtained at room temperature by using a Nicolet 6700 spectrometer. XRD patterns were recorded using CuKα radiation and a X‐ray diffractometer from Bruker. SEM images were collected using a SU8010 scanning electron microscope at a voltage of 5 kV. Zeta potentials were obtained using a Zen3600 Zetasizer Nano ZS. XPS spectra were obtained using a Thermo Scientific ESCALAB 250 Xi with a mo56nochromatic Al‐Kα X‐ray source. High‐resolution transmission electron microscope (HR‐TEM) images were obtained using a FEI Tecnai G2 F30 instrument and an acceleration voltage of 300 kV. AFM images were obtained by using a Bruker Dimension Icon. The digital bridge (LCR) meter (Keysight E5071C) was used for dielectric constant measurements. Testing was conducted in a temperature‐controlled environment using a parallel‐plate electrode system.

## Author Contributions

Liang Ma: conceptualization, data curation, formal analysis, methodology, software, and writing – original draft. Mengdi Liu: methodology and project administration. Yuxi Yang: data curation and software. Zehao Wang: data processing. Lan Shi: reviewing and editing. Limin Wu: funding acquisition, reviewing, and editing.

## Conflicts of Interest

The authors declare no conflicts of interest.

## Supporting information




**Supporting File 1**: advs74225‐sup‐0001‐SuppMat.docx.

**Supporting File 2**: advs74225‐sup‐0002‐VideoS1.mp4.

**Supporting File 3**: advs74225‐sup‐0003‐VideoS2.mp4.

**Supporting File 4**: advs74225‐sup‐0004‐VideoS3.mp4.

## Data Availability

The data that support the findings of this study are available from the corresponding author upon reasonable request.
