# Supplementary material for: Smart Energy–Harvesting Coating for Moisture–Droplets Based on Ionic Diodes and Transistor–Like Structures
Source: Adv Sci (Weinh). 2026 Feb 4;13(21):e21476. doi: 10.1002/advs.202521476 (PMC13073254; doi:10.1002/advs.202521476)
Supplement: Supplementary file 1 — Supporting File 1: advs74225‐sup‐0001‐SuppMat.docx. [file ADVS-13-e21476-s002.docx]

**Smart Energy Harvesting Coating for Moisture-Droplets Based on Ionic Diodes and Transistor-Like Structures**

*Liang Ma, ^a^ Mengdi Liu, ^a^ Yuxi Yang, ^a^ Zehao Wang, ^a^ Lan Shi, ^a^* Limin Wu ^a^**

*^a^* College of Smart Materials and Future Energy, State Key Laboratory of Coatings for Advanced Equipment, Advanced Coatings Research Center of Ministry of Education of China, Fudan University, Shanghai 200433, China.


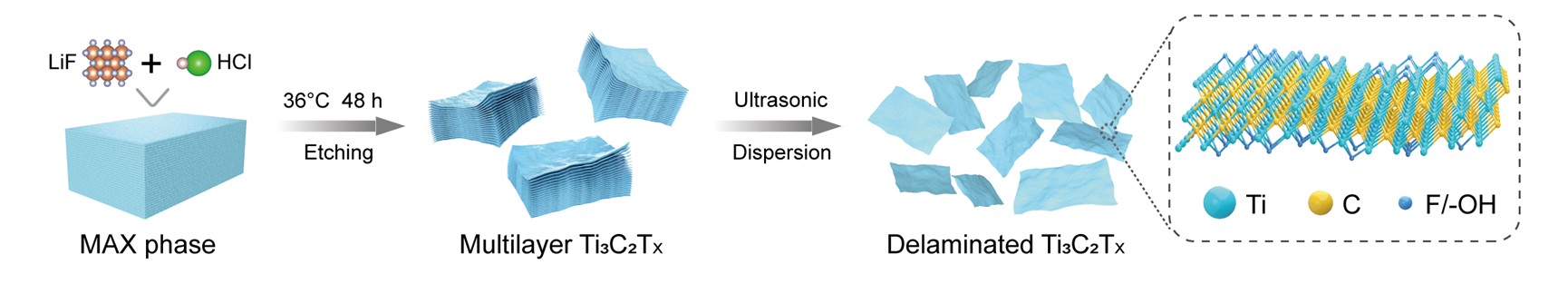


**Figure S1.** Schematic diagram of the MILD process for preparing monolayer MXene.


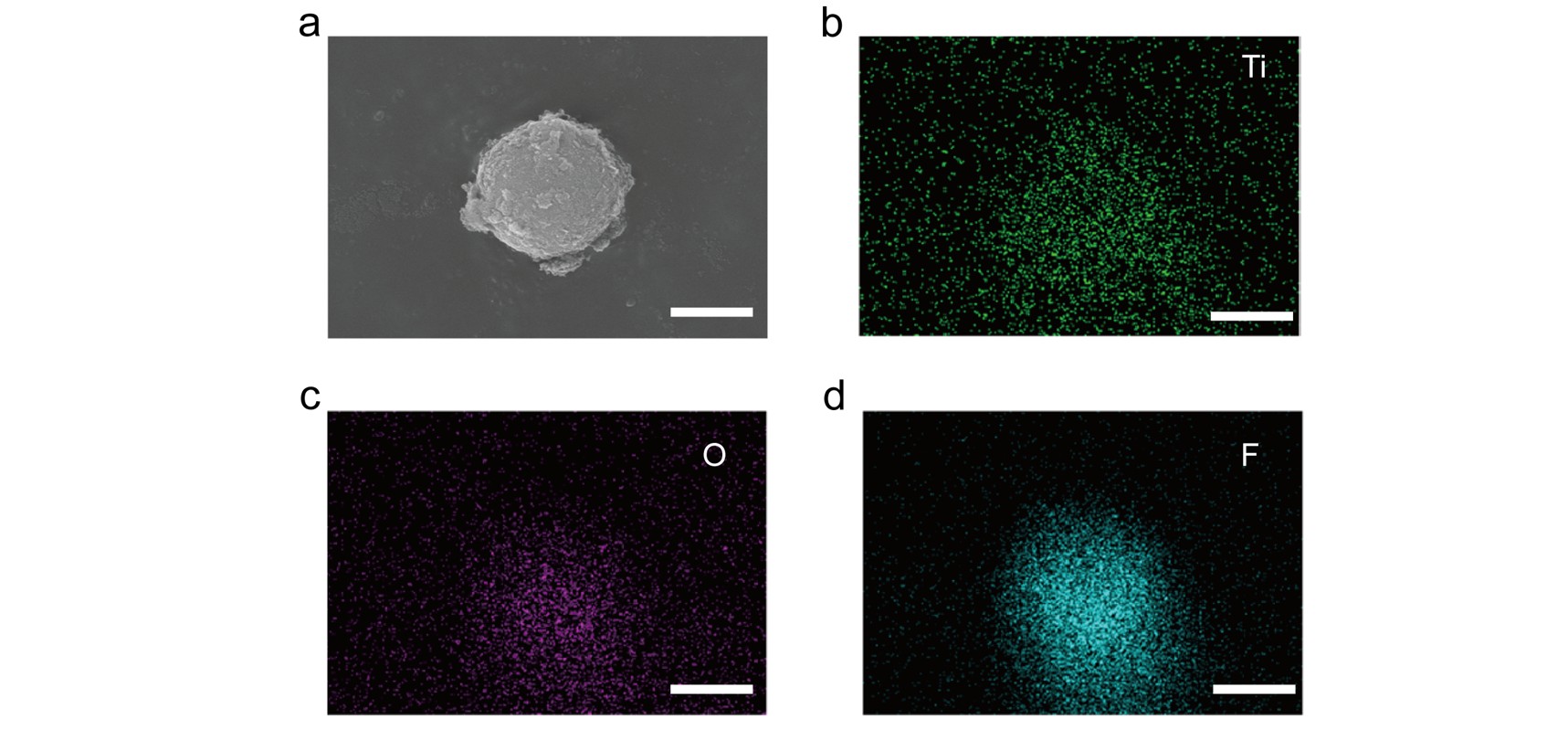


**Figure S2.** EDS characterization results of MGM microspheres. (a) MGM microspheres, (b) Ti element distribution, (c) O element distribution, (d) F element distribution.


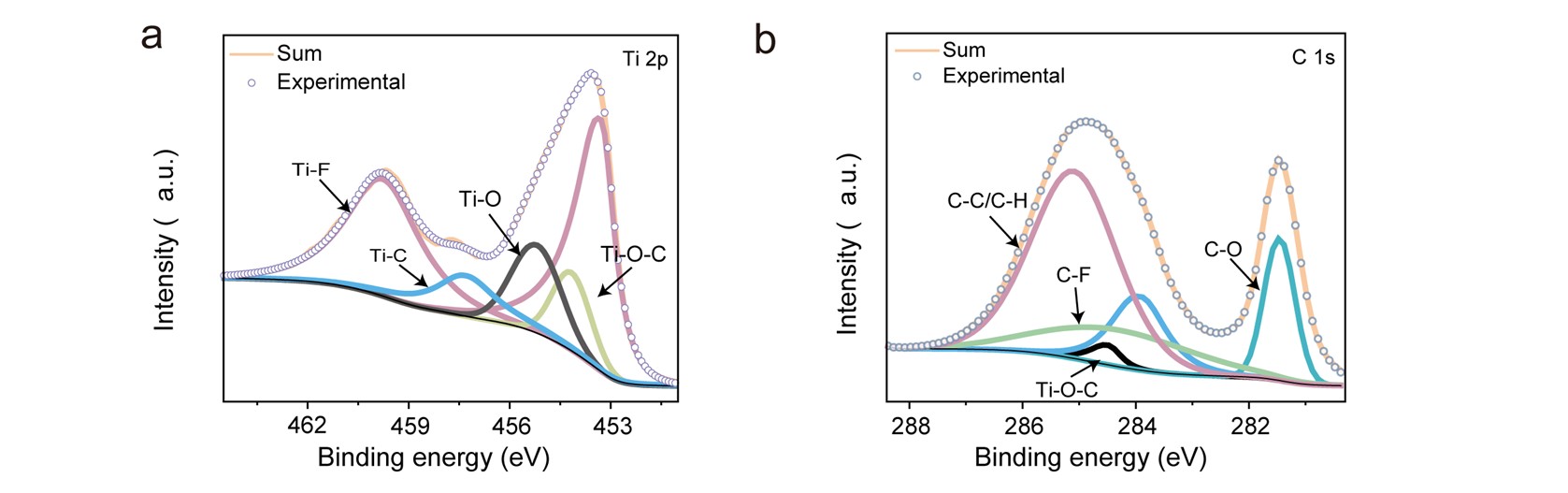


**Figure S3.** XPS characterization results for MGM. (a) Ti 2p peak diagram, (b) C 1s peak diagram.


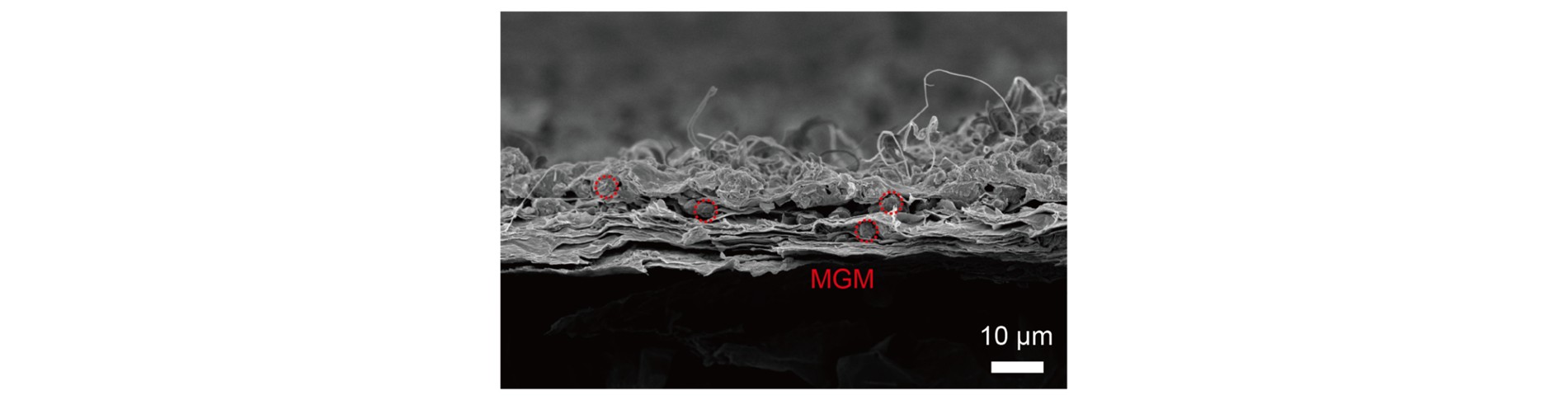


**Figure S4.** Cross-sectional SEM image of MMGM coating.


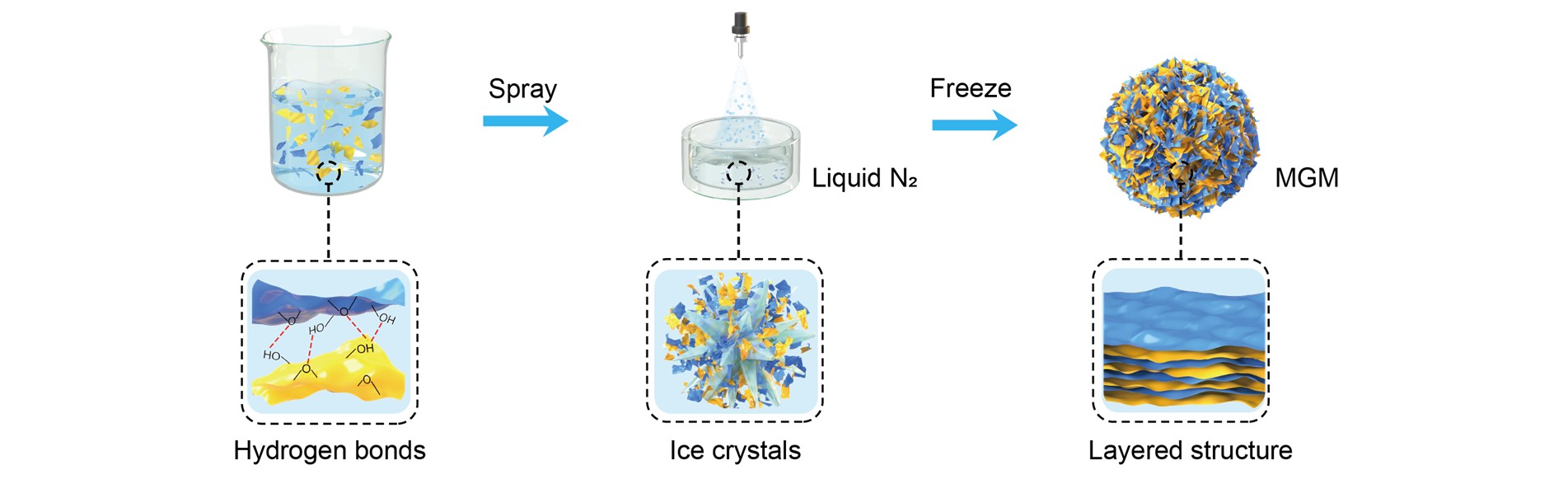


**Figure S5.** Preparation process and structural schematic diagram of aerogel microsphere-reinforced materials.


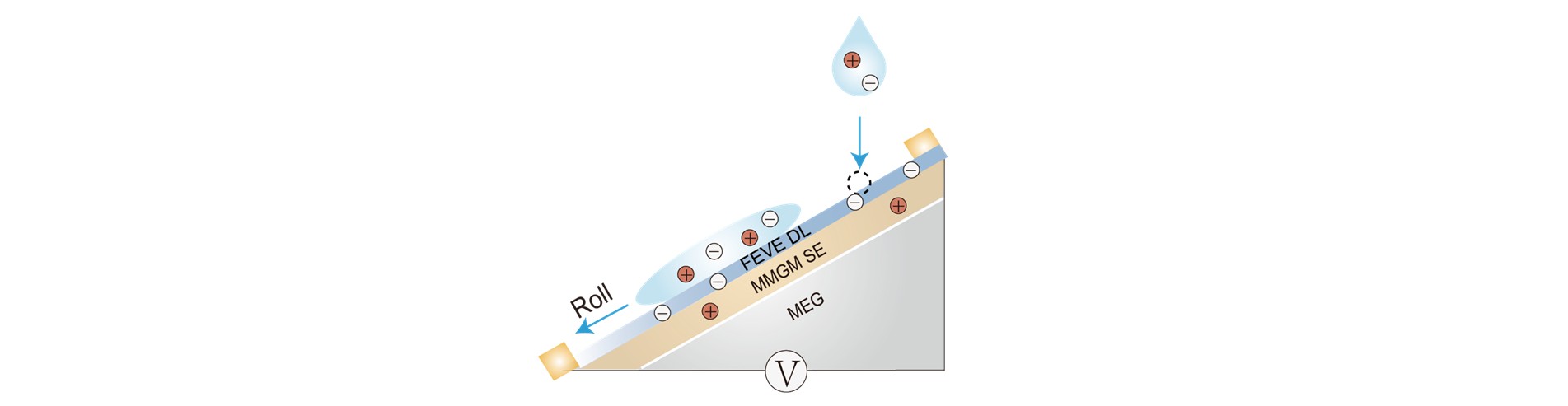


**Figure S6.** Schematic diagram of charge distribution in the system during solid-liquid contact of droplet**s**


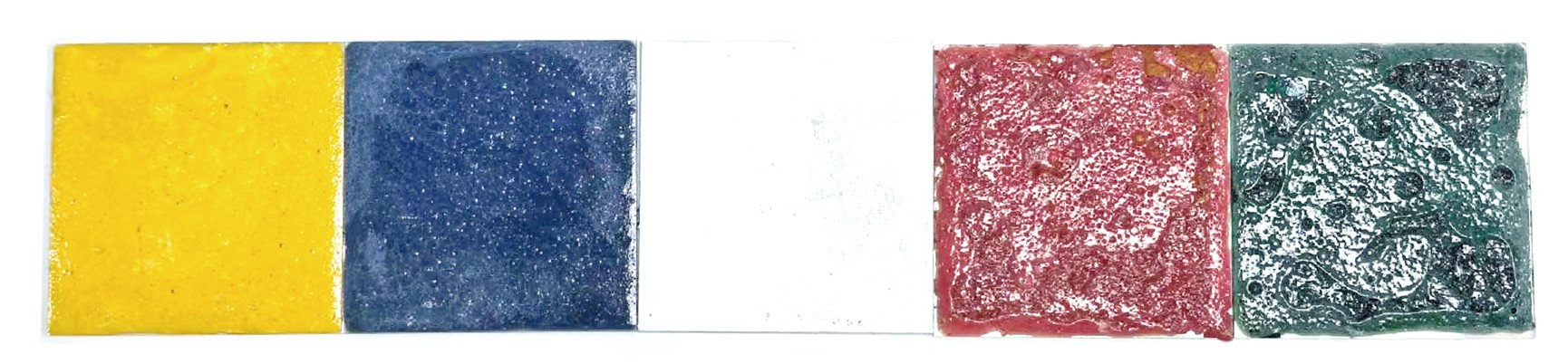


**Figure S7.** Dielectric layers prepared by adding different colored pigments.


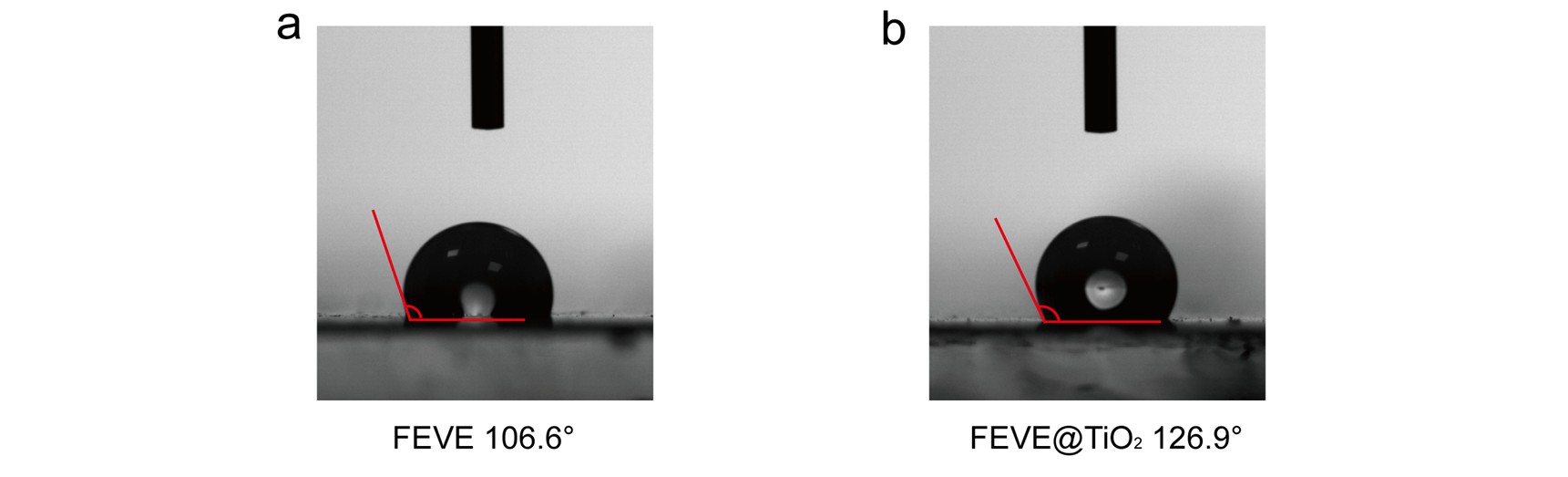


**Figure S8.** Hydrophobicity of the DEG dielectric layer. (a) Hydrophobicity of FEVE layer, (b) Hydrophobicity of FEVE@TiO₂ layer.


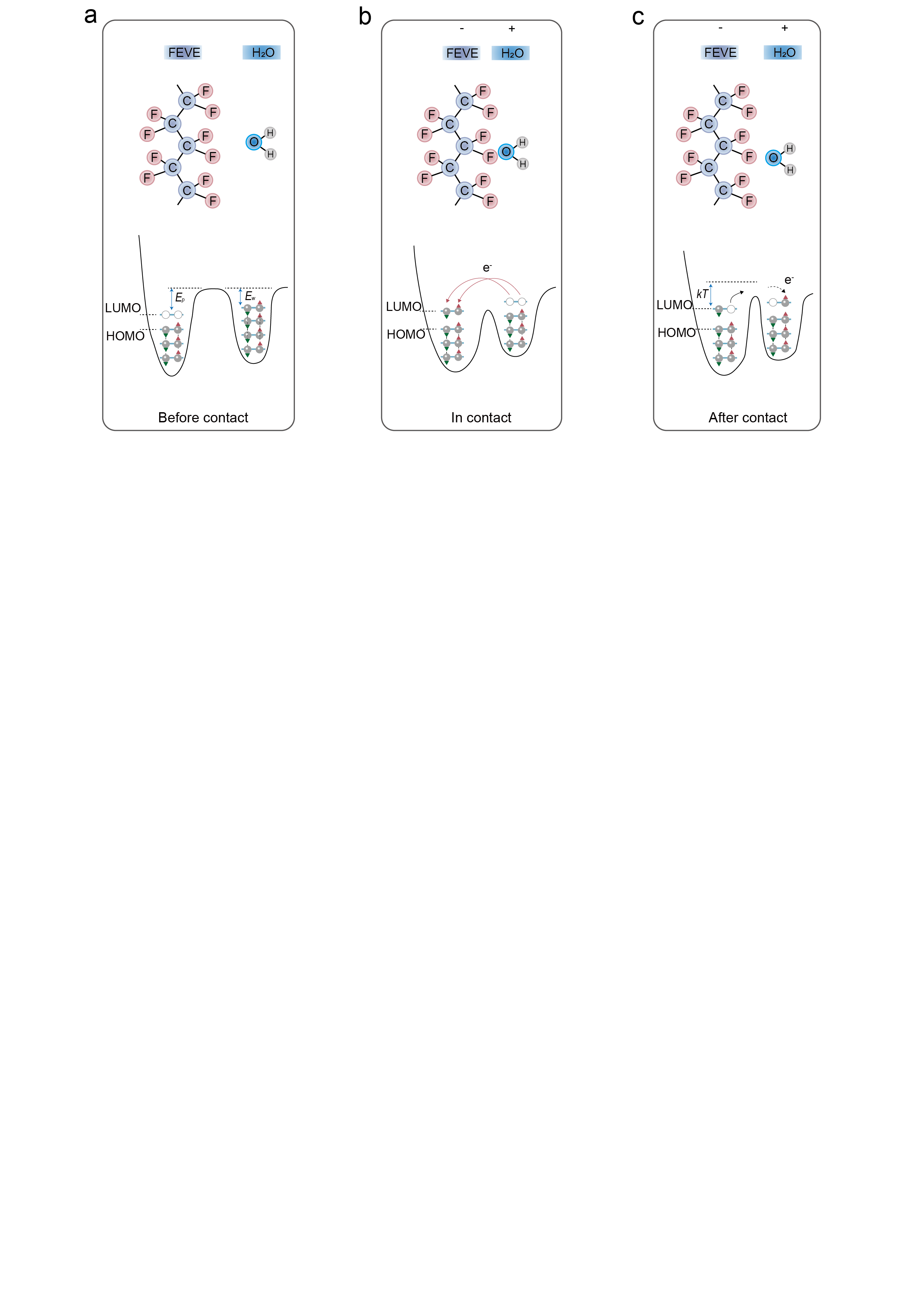


**Figure S9.** The electrons transfer between the molecule of water and PTFE. (a) Non-contact state, (b) Contact state, (c) Disconnected state

The mechanism of charge transfer at the molecular level between water molecules and polymer molecules can be explained through the following process. The outermost electrons of the oxygen atom in a water molecule form an electron cloud, which is attracted by the lowest unoccupied molecular orbital (LUMO) on the FEVE surface. Therefore, during contact electrochemistry between water droplets and the FEVE surface, the LUMO is regarded as an electron acceptor. As shown in Figure S9a, the escape energies of the LUMO electrons in FEVE and the oxygen atom electrons in the water molecule can be denoted as E_p_ and E_w_, respectively. When water molecules contact the PTFE surface, electrons from the oxygen atom in the water molecule can migrate to the LUMO of FEVE, as shown in Figure S9b.

When the two materials separate, as depicted in Figure S9c, if the Ep (energy barrier) of the FEVE layer exceeds the thermal energy fluctuation (kT), electrons that jump to FEVE will primarily remain trapped within the LUMO. As temperature (T) increases, raising the electron thermal energy (kT), electrons can more readily escape the potential well. Water molecules exhibit a tendency to polarize; due to their positive charge, adjacent water molecules also become polarized. Consequently, electrostatic energy arises from the interaction between the FEVE surface and water droplets.


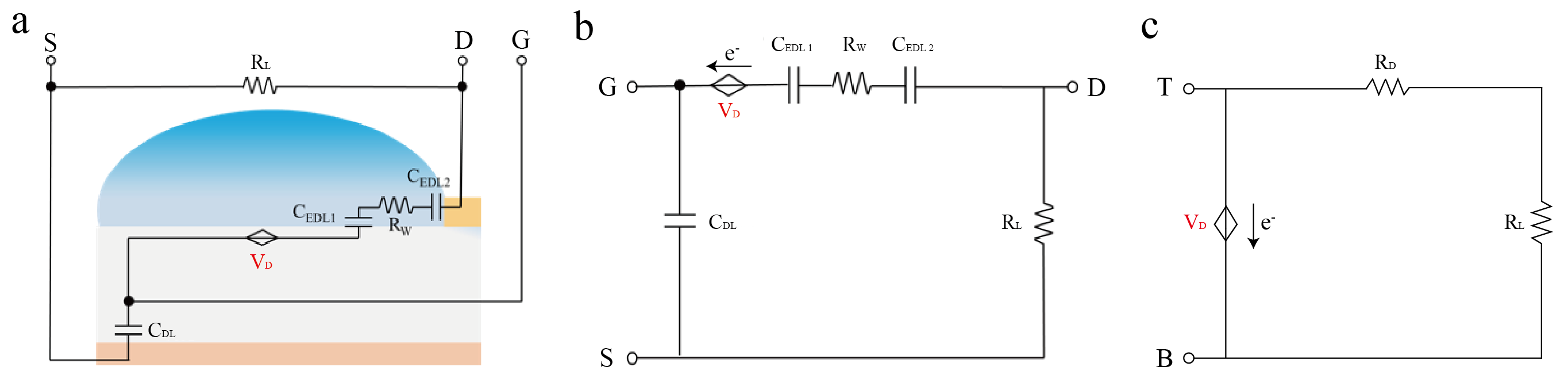


**Figure S10.** Working mechanism and equivalent circuit. (a) Equivalent electronic components in the DEG. (b) Equivalent circuit model of DEG. (c) Equivalent circuit model of MEG.

As shown in Figure S10a, to clarify the droplet nanogenerator DEG testing and energy harvesting process, each key component of the DEG is treated as a port. The dielectric layer (DL), carrying plenty of negative charges, is defined as port G, while the bottom electrode serves as port S. The potential difference between ports G and S is defined as the droplet voltage (V_D_). Driven by V_D_, positive ions in the droplet are attracted to the dielectric layer surface, forming an electric double layer (EDL). When the diffusing droplet contacts the top electrode (port D), a droplet channel forms between D and S, constituting a closed system. Under these conditions, charge transfers from port S to D under V_D_ drive, generating electrical power output. Consequently, we can evaluate relevant DEG output performance through electrical signals between ports D and S. As shown in Figure S10b, we constructed a circuit model connecting the DEG's three ports to three interfacial capacitances (C_DL_, C_EDL1_, and C_EDL2_), two impedances (water impedance R_W_ and load impedance R_L_), and a voltage source V_D_. Since the droplet contact-separation process generates alternating current, the figure indicates the direction of electron transfer flow at the instant of droplet-drain electrode contact.

Figure S10c illustrates the equivalent circuit diagram and electron transfer flow direction of the MEG process. The top electrode is defined as port “T,” the bottom electrode as port “B,” and the system includes two impedances (water impedance R_W_ and load impedance R_L_). The potential difference between ports T and B is defined as moisture voltage V_D_. During the testing process, the electrodes were grounded.


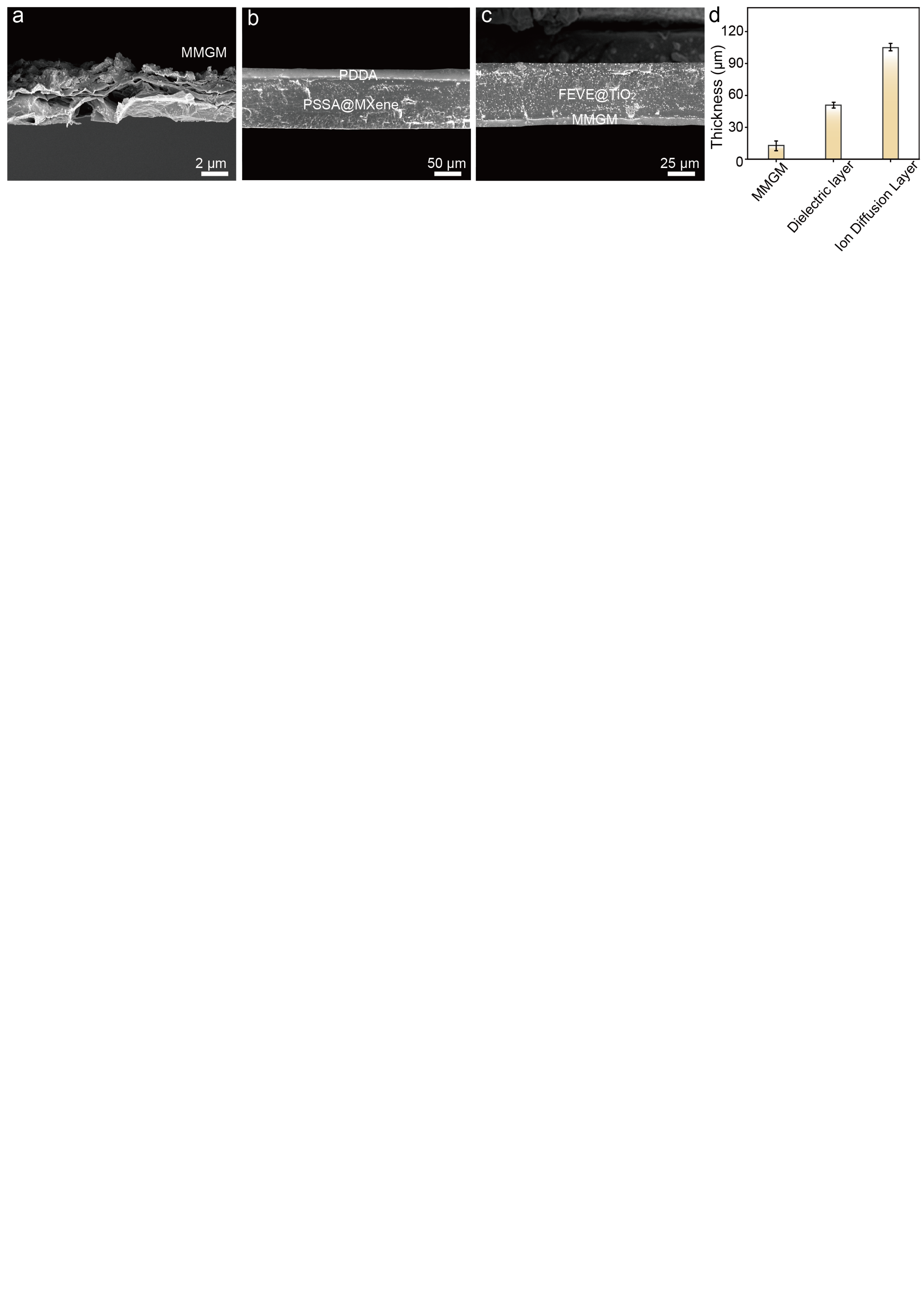


**Figure S11.** Analysis of coating morphology and thickness uniformity across functional layers. (a) Cross-section of electrode layer, (b) Cross-sectional morphology of ion diffusion layer, (c) Cross-sectional morphology of dielectric layer and electrode, (d) Statistical analysis of thickness uniformity across coatings (10 data points).


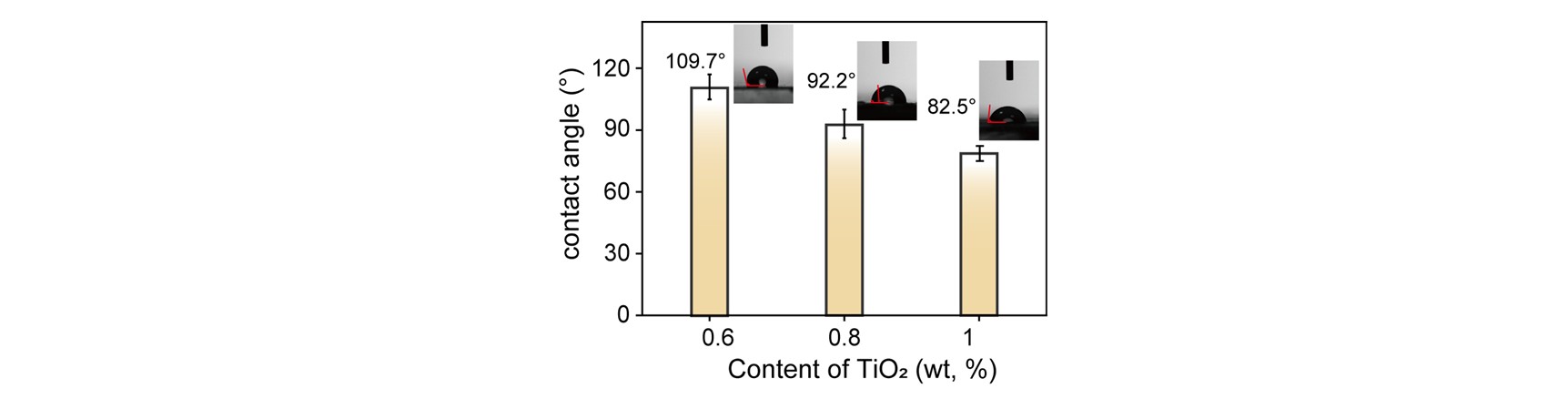


**Figure S12.** Effect of TiO₂ content on hydrophobicity.


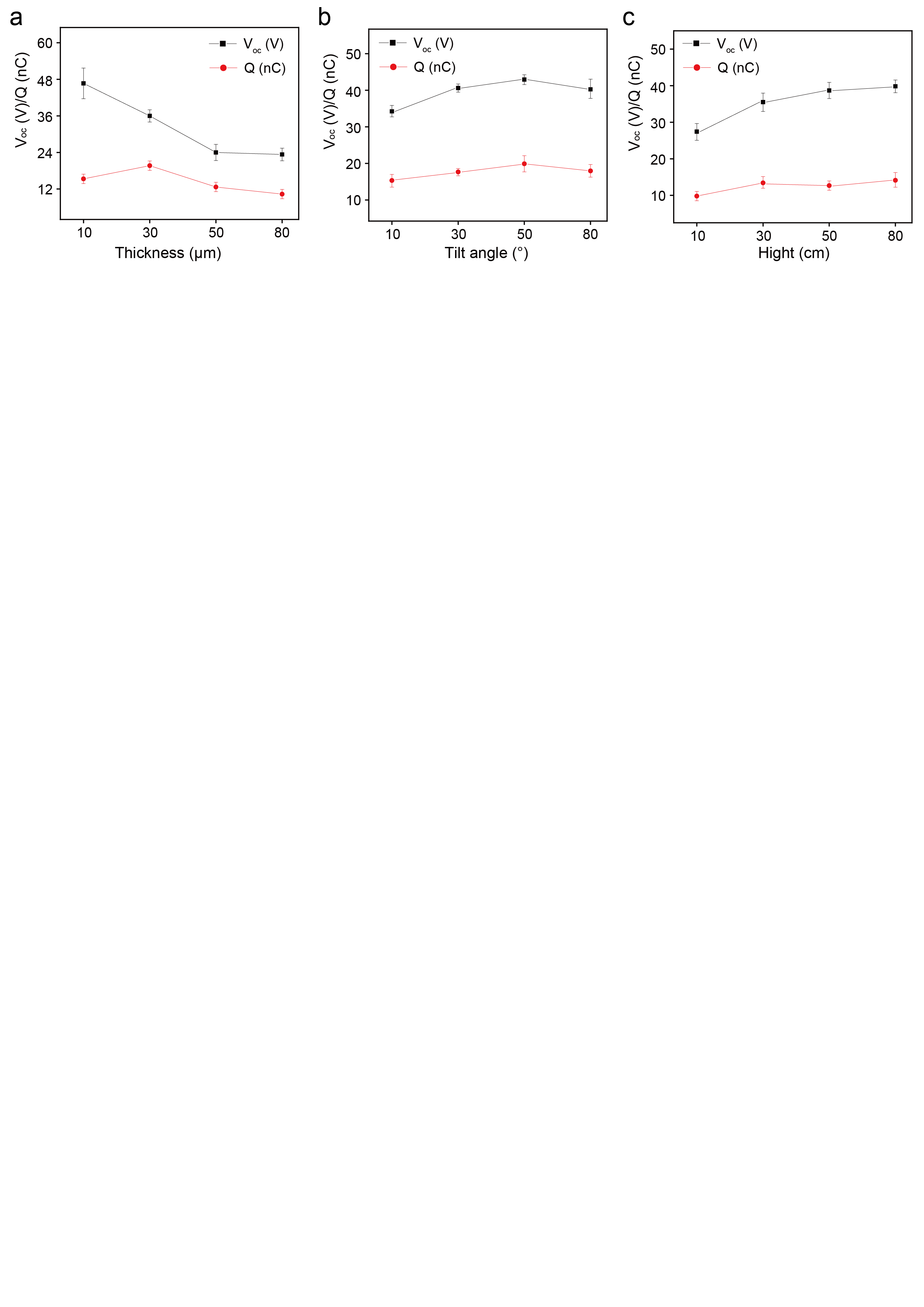


**Figure S13.** Effects of dielectric layer thickness (a), film tilt angle (b), and liquid drop height (c) on output performance.


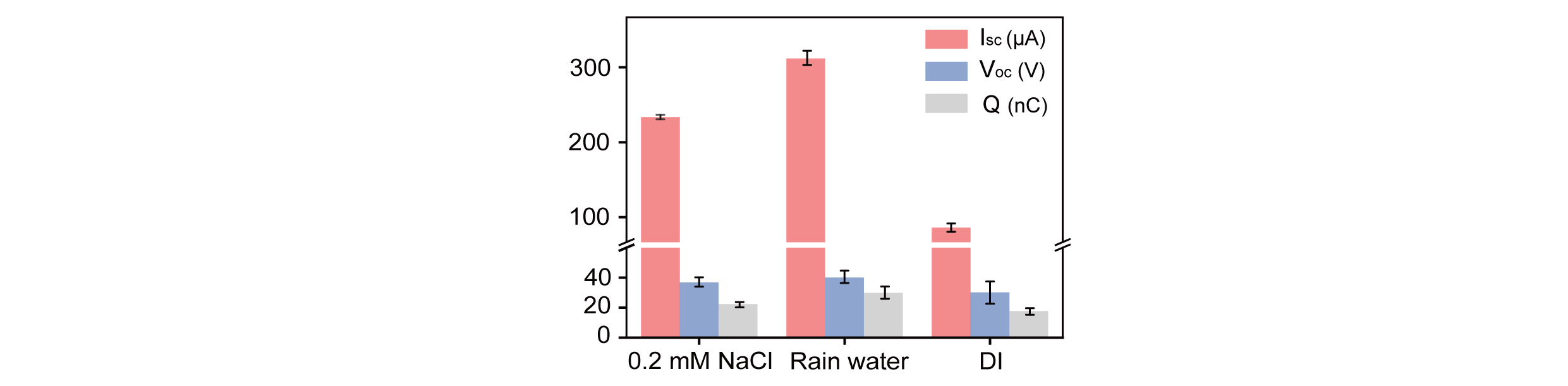


**Figure S14.** Comparison of the effect of different droplet types on DEG output performance.

**Figure S15.** Effect of droplet pH on the output performance of DEG. (a) Effect on short-circuit current, (b) Effect on open-circuit voltage, (c) Effect on charge transfer.

The pH value of the droplet affects the contact charge during its interaction with the dielectric surface. If the droplet's pH exceeds 3, it becomes positively charged, while the dielectric material surface (FEVE) carries a negative charge, leading to an increase in contact charge. Conversely, if the droplet's pH is below 3, it becomes negatively charged, and the dielectric material surface carries a positive charge. ^[1]^ We supplemented the effects of droplet pH on DEG performance. It can be observed that as pH increases, contact charge initially rises due to enhanced ion transfer during contact charge generation, leading to improved output performance.


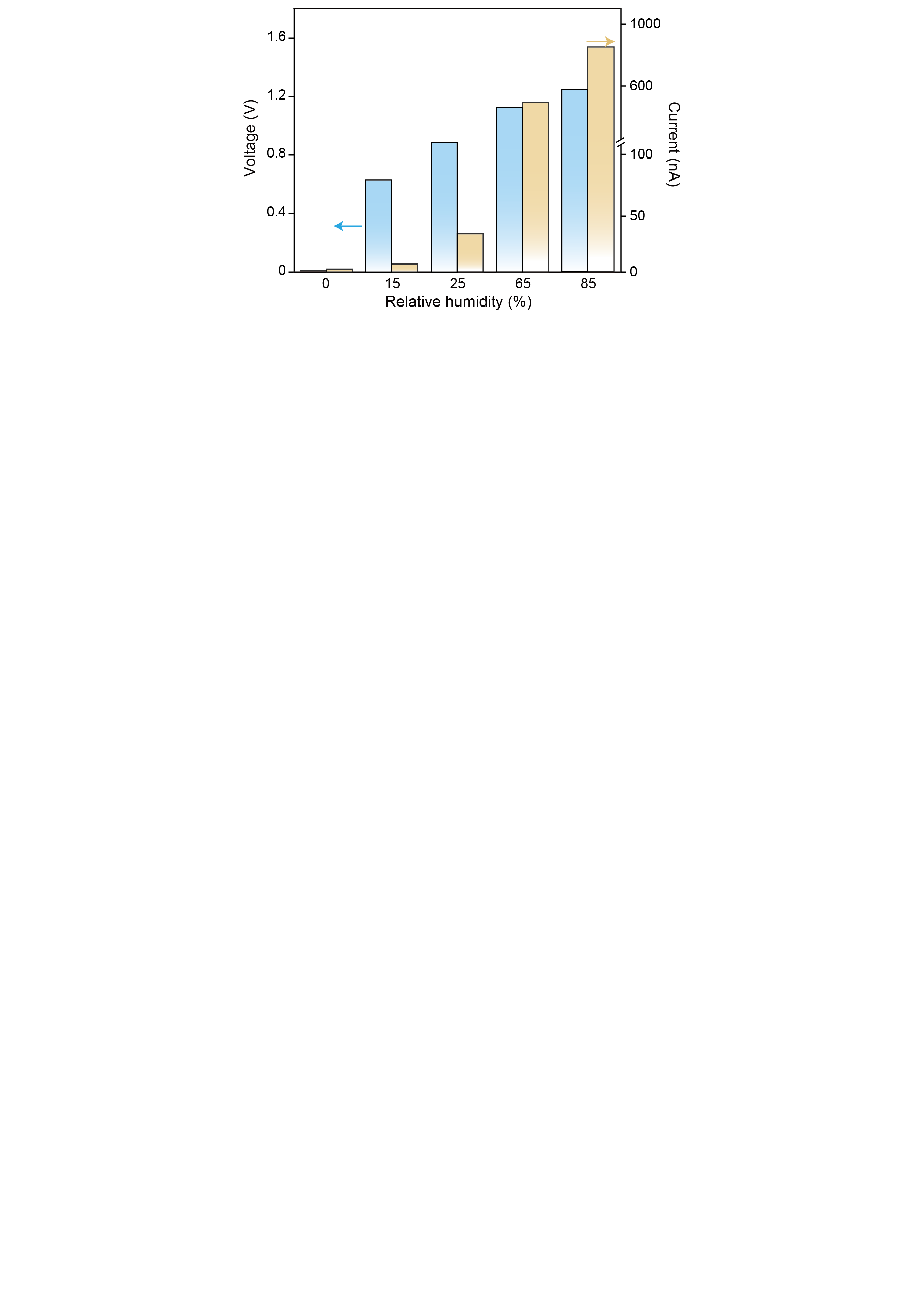


**Figure S16.** Voltage and current output in response to variation in RH (25 °C) under atmospheric conditions.

**Figure S17.** Voltage and current output in response to variation in temperature under atmospheric conditions (25% RH).


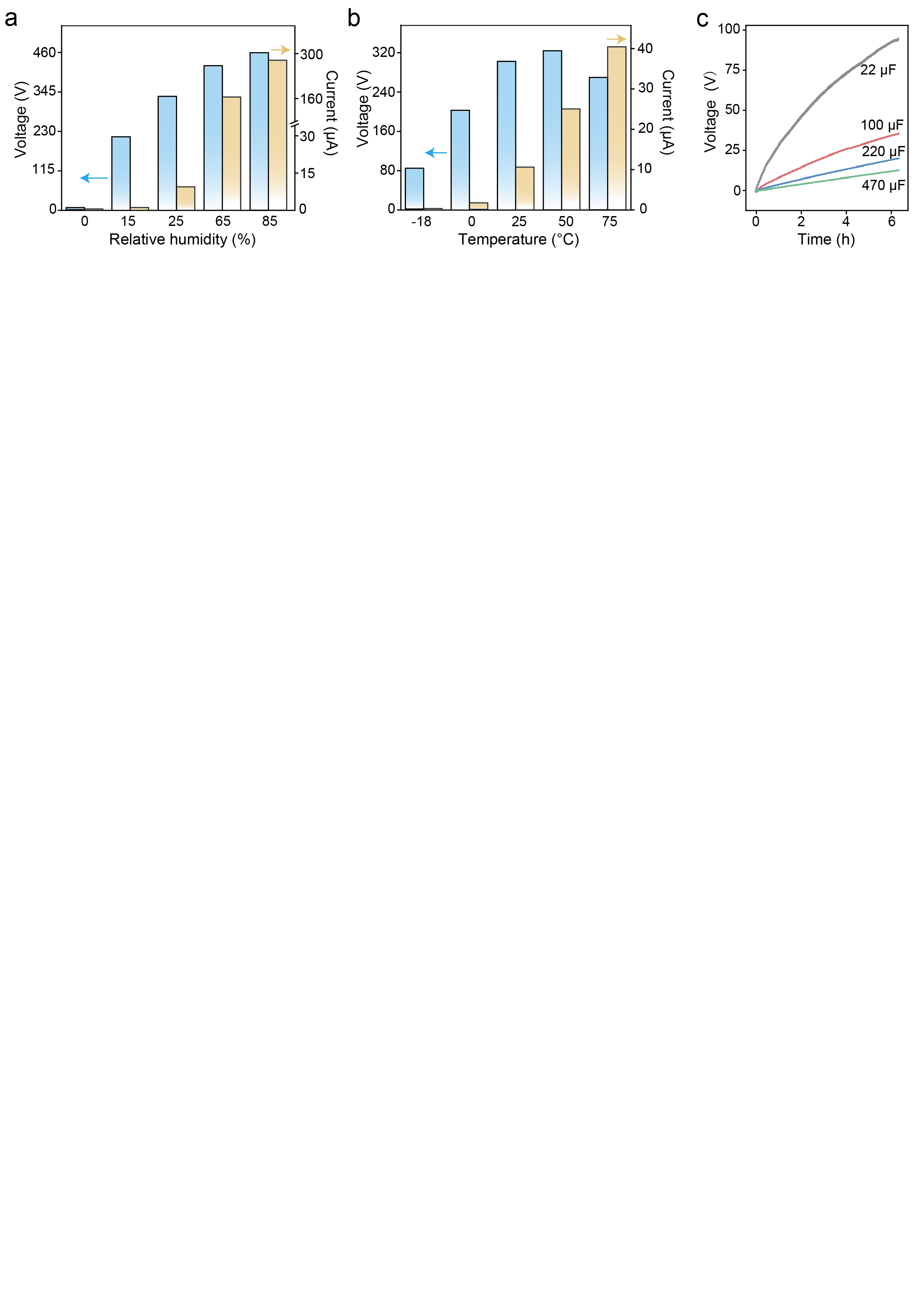


**Figure S18.** Effect of temperature and humidity on integrated panels and charging performance. (a) Effect of humidity on integrated coatings, (b) Effect of temperature on integrated coatings, (c) Capacitor charging performance.


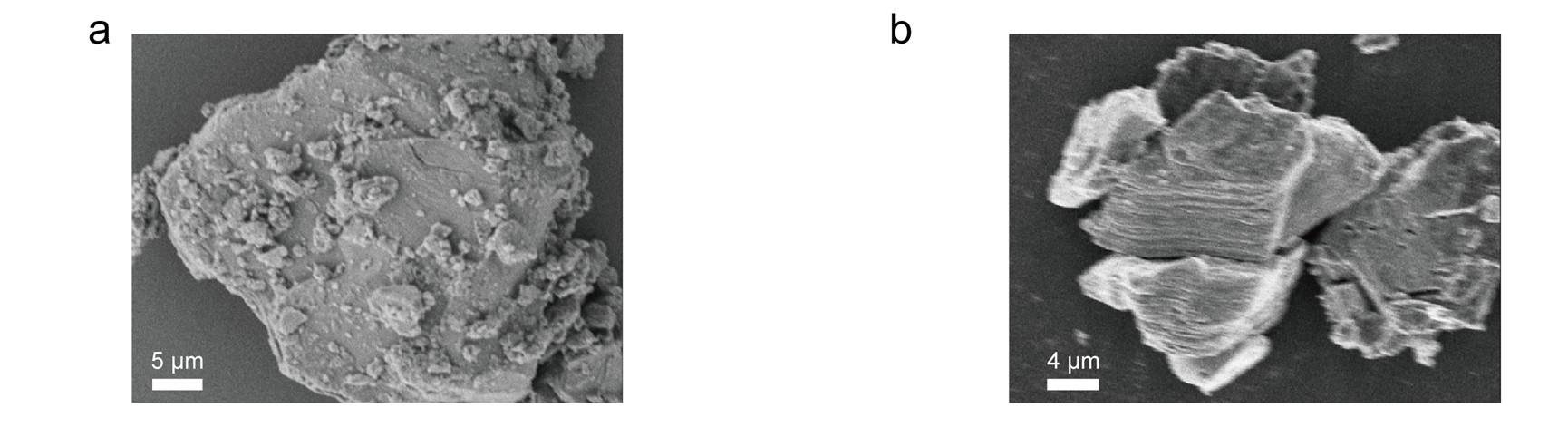


**Figure S19.** SEM images of MAX (a) and MXene (b).


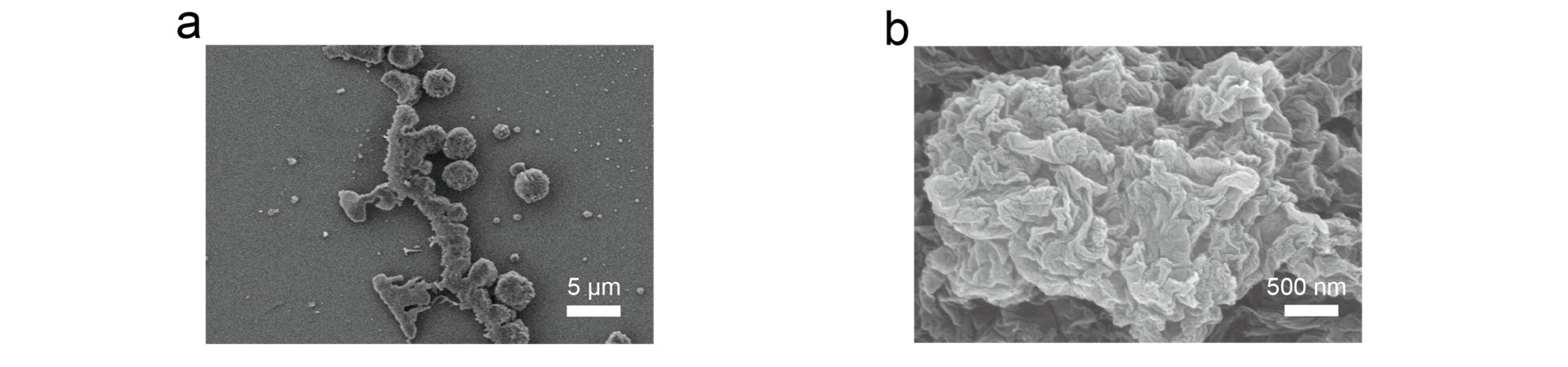


**Figure S20.** Morphology of MGM microspheres (a) and magnified view (b).


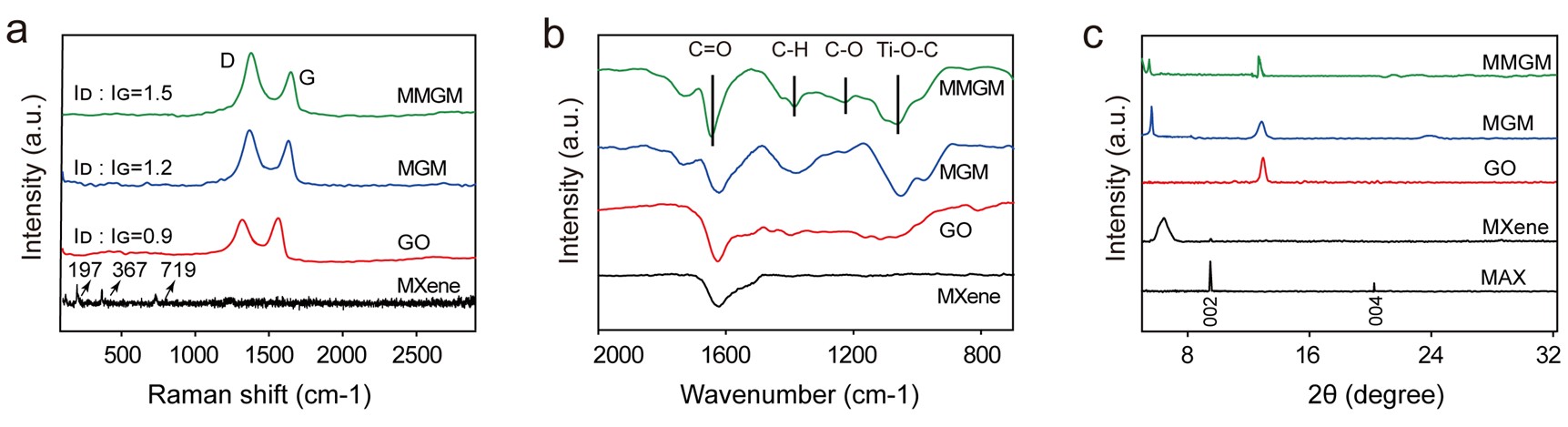


**Figure S21.** Characterization of the experimentally synthesized material. (a) Raman spectrum, (b) FTIR spectrum, (c) XRD spectrum.


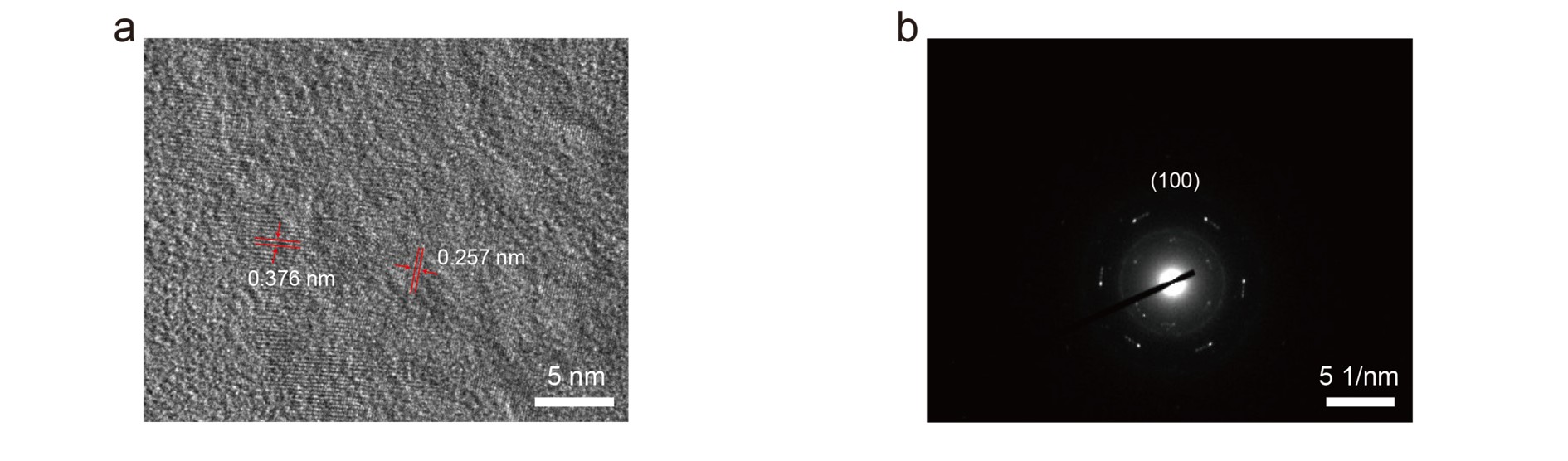


**Figure S22.** TEM images of MXene-GO hybrid nanosheets. (a) HRTEM image of MXene loaded on GO, (b) SAED image of MXene loaded on GO.


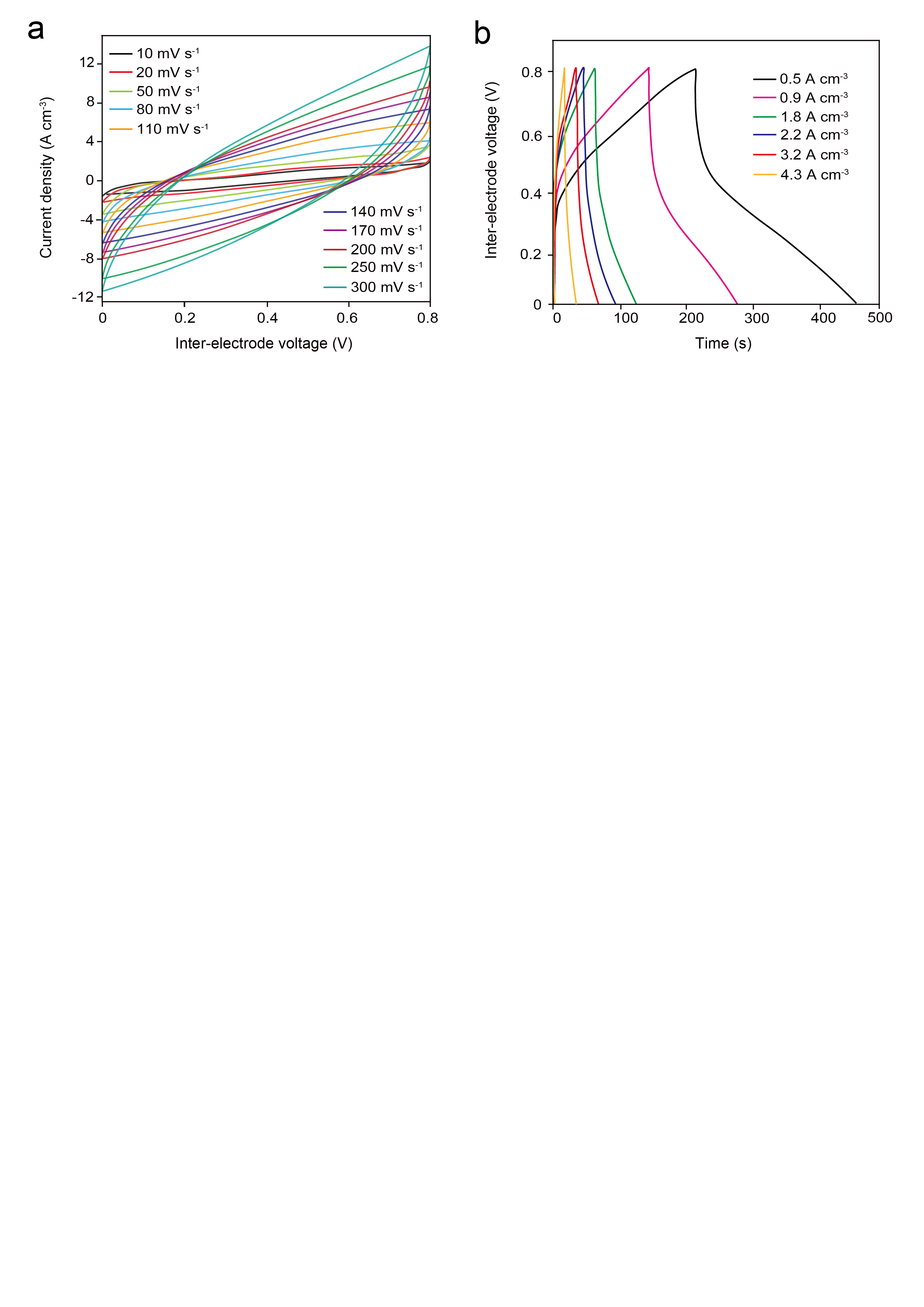


**Figure S23.** Structural Integrity Electrochemical Testing. (a) Cyclic voltammetry (CV) curves at scan rates of 10 to 300 mV s^−1^ for the MMGM-PDMS coating system. (b) GCD curves for the MMGM-PDMS coating system.

**Table S1.** Comparison of common dielectric material properties

| **Materials** | **Dielectric constant (εᵣ)** | **Thermal Stability**  **(°C)** | **Cost** | **Main Application Scenarios** |
| --- | --- | --- | --- | --- |
| **TiO_2_** | 80-110 | >600 | Moderate | Coatings |
| **Al_2_O_3_** | 8-10 | >1500 | Low | Encapsulation material |
| **SiO_2_** | 3.9 | >1600 | Low | Microelectronics |
| **BN** | 4-5 | >900 | High | Thermal interface material |

**Table S2.** Summary of related performance parameters of MEG at low RH.

| **Materials** | **RH/∆RH**  **（%）** | **Voltage**  **（°C）** | **Peak volumetric power density**  **（μW cm^-^³）** | **Reference** |
| --- | --- | --- | --- | --- |
| **Protein nanowire** | 35 | 0.32 | 1.2 | [2] |
| **Graphene oxide** | 25 | 0.20 | 0.9 | [3] |
| **Gradient graphene**  **oxide and graphene oxide** | 20 | 0.52 | 1.6 | [4] |
| **TiO_2_ nanowire** | 40 | 0.07 | 0.7 | [5] |
| **Gradient**  **polypyrrole foam** | 27 | 0.03 | 0.4 | [6] |
| **Polyelectrolyte film** | 25 | 0.85 | 1.1 | This work |

**Table S3.** Voltage output of the integrated device for water related power generators.

| **NO.** | **Material** | **Voltage (V)** | **Serial number** | **Integrated voltage (V)** | **Ref.** |
| --- | --- | --- | --- | --- | --- |
| **1** | Carbon black film | 1.2 | 4 | 4.8 | [7] |
| **2** | Printable carbon film | 1 | 5 | 4.2 | [8] |
| **3** | Solid oxides) | 2.5 | 6 | 14.8 | [9] |
| **4** | Fabric coated with carbon black | 0.5 | 4 | 1.67 | [10] |
| **5** | Wet textile | 0.7 | 40 | 12 | [11] |
| **6** | Ni-Al layered double hydroxide | 0.7 | 3 | 2.2 | [12] |
| **7** | Ni-Al layered double hydroxide with tailored structure | 0.6 | 4 | 2.5 | [13] |
| **8** | Stacked hydrogels | 0.15 | 612 | 110 | [14] |
| **9** | Graphene oxide and sodium  polyacrylate | 0.4 | 68 | 26.2 | [15] |
| **11** | MMGM coating electrode /PSSA/PDDA | 0.85 | 360 | 300 | This work |

**Reference**

[1] M. D. Sosa, M. L. Martínez Ricci, L. L. Missoni, et al., "Liquid–polymer triboelectricity: chemical mechanisms in the contact electrification process", *Soft Matter* **2020**, *16*, 7040.<http://10.1039/D0SM00738B>

[2] X. Liu, H. Gao, J. E. Ward, et al., "Power generation from ambient humidity using protein nanowires", *Nature* **2020**, *578*, 550.<http://10.1038/s41586-020-2010-9>

[3] H. Cheng, Y. Huang, F. Zhao, et al., "Spontaneous power source in ambient air of a well-directionally reduced graphene oxide bulk", *Energ. Environ. Sci.* **2018**, *11*, 2839.<http://10.1039/C8EE01502C>

[4] Y. Huang, H. Cheng, C. Yang, et al., "Interface-mediated hygroelectric generator with an output voltage approaching 1.5 volts", *Nat. Commun.* **2018**, *9*, 4166.<http://10.1038/s41467-018-06633-z>

[5] D. Shen, M. Xiao, G. Zou, et al., "Self-Powered Wearable Electronics Based on Moisture Enabled Electricity Generation", *Adv. Mater.* **2018**, *30*, 1705925.<http://https://doi.org/10.1002/adma.201705925>

[6] S. Lee, J. Eun, S. Jeon, "Facile fabrication of a highly efficient moisture-driven power generator using laser-induced graphitization under ambient conditions", *Nano Energy* **2020**, *68*, 104364.<http://https://doi.org/10.1016/j.nanoen.2019.104364>

[7] W. Guo, Y. Tian, L. Jiang, "Asymmetric Ion Transport through Ion-Channel-Mimetic Solid-State Nanopores", *Acc. Chem. Res.* **2013**, *46*, 2834.<http://10.1021/ar400024p>

[8] G. Xue, Y. Xu, T. Ding, et al., "Water-evaporation-induced electricity with nanostructured carbon materials", *Nat. Nanotechnol.* **2017**, *12*, 317.<http://10.1038/nnano.2016.300>

[9] C. Shao, B. Ji, T. Xu, et al., "Large-Scale Production of Flexible, High-Voltage Hydroelectric Films Based on Solid Oxides", *ACS Appl. Mater. Interfaces* **2019**, *11*, 30927.<http://10.1021/acsami.9b09582>

[10] T. G. Yun, J. Bae, A. Rothschild, et al., "Transpiration Driven Electrokinetic Power Generator", *ACS Nano* **2019**, *13*, 12703.<http://10.1021/acsnano.9b04375>

[11] S. S. Das, V. M. Pedireddi, A. Bandopadhyay, et al., "Electrical Power Generation from Wet Textile Mediated by Spontaneous Nanoscale Evaporation", *Nano Lett.* **2019**, *19*, 7191.<http://10.1021/acs.nanolett.9b02783>

[12] J. Sun, P. Li, J. Qu, et al., "Electricity generation from a Ni-Al layered double hydroxide-based flexible generator driven by natural water evaporation", *Nano Energy* **2019**, *57*, 269.<http://https://doi.org/10.1016/j.nanoen.2018.12.042>

[13] J. Tian, Y. Zang, J. Sun, et al., "Surface charge density-dependent performance of Ni–Al layered double hydroxide-based flexible self-powered generators driven by natural water evaporation", *Nano Energy* **2020**, *70*, 104502.<http://https://doi.org/10.1016/j.nanoen.2020.104502>

[14] T. B. H. Schroeder, A. Guha, A. Lamoureux, et al., "An electric-eel-inspired soft power source from stacked hydrogels", *Nature* **2017**, *552*, 214.<http://10.1038/nature24670>

[15] Y. Huang, H. Cheng, C. Yang, et al., "All-region-applicable, continuous power supply of graphene oxide composite", *Energ. Environ. Sci.* **2019**, *12*, 1848.<http://10.1039/C9EE00838A>
